# The Impact of the Eligibility Threshold of a French Means‐Tested Health Insurance Programme on Doctor Visits: A Regression Discontinuity Analysis

**DOI:** 10.1002/hec.3464

**Published:** 2017-03-20

**Authors:** Sophie Guthmuller, Jérôme Wittwer

**Affiliations:** ^1^ PSL, Université Paris Dauphine LEDa‐LEGOS Paris France; ^2^ European Commission Joint Research Centre Ispra (VA) Italy; ^3^ Université de Bordeaux Inserm U1219 Bordeaux Population Health Bordeaux France

**Keywords:** public health insurance, means‐tested social programme, doctor visits, regression discontinuity design, France

## Abstract

This paper assesses the impact of eligibility for a free means‐tested complementary health insurance plan, called Couverture Maladie Universelle Complémentaire (CMUC), on doctor visits. We use information on the selection rule to qualify for the plan to identify the effect of eligibility and adopt a regression discontinuity approach. Our sample consists of low‐income individuals enrolled in the Health Insurance Fund and recipients of social benefits from the Family Allowance Fund of an urban area in Northern France. Our findings do not show significant impacts of the CMUC threshold on the number of doctor visits within the full sample. Among the subsample of adults under 30 years old, however, eligible individuals are more likely to see a specialist and have, on average, significantly more specialist visits than non‐eligible individuals. This specific impact of the CMUC cut‐off point among young adults may be explained by the fact that young adults are less likely to be covered by a complementary health insurance plan when they are not recipients of the CMUC plan. © 2017 The Authors. *Health Economics* Published by John Wiley & Sons, Ltd.

## 1 Introduction

In France, a free complementary health insurance (CHI) plan called Couverture Maladie Universelle Complémentaire (CMUC) pays most out‐of‐pocket expenses for the poorest citizens. This means‐tested plan was introduced to remove financial barriers to access to CHI and thus healthcare.

Since its introduction in 2000, it has been widely shown that CMUC recipients have gained improved access to care and that their healthcare use has drawn close to that of individuals covered by a CHI plan, despite the refusal of some doctors to treat CMUC patients (Raynaud, 2003; Grignon and Perronnin, 2003; Boisguérin, 2009; Desprès, 2010).

There is evidence that CMUC has largely overcome the deficiencies of the French healthcare system in terms of financial access to care among the poorest citizens (Ricci, 2011; Grignon *et al*., 2008). However, little is known about poor individuals who are not eligible for the free plan. In fact, individuals with an income just above the CMUC threshold are poorly covered by a CHI plan, despite the 2004 introduction of a voucher (called Aide Complémentaire Santé, ACS) to financially help individuals with an income up to 35% (since January 2013) above the CMUC cut‐off point to purchase a CHI plan (Guthmuller *et al*., 2014). Access to primary care thus remains expensive for those individuals (Jusot and Wittwer, 2009; Perronnin *et al*., 2011).

As in many social programmes for which access is conditional on income, the impact of the chosen threshold is an issue for public policy decision making. When designing such a programme, one has to decide on the generosity of the coverage and to know whether extending or reducing the target population marginally will have effects; namely determine healthcare utilisation of those at the margin of eligibility. One must then be very careful in setting the cut‐off point between the recipients and the non‐recipients because the chosen cut‐off point may have adverse effects and may hit those who are in some sense the most vulnerable group.

Numerous recent papers in the United States have highlighted this problem in evaluating the impact of the Affordable Care Act on enrolment and healthcare utilisation. The Affordable Care Act introduced changes in eligibility rules, coverage and enrolment processes to existing health insurance programmes, particularly for Medicaid (De la Mata, 2012; Sommers *et al*., 2013; Dague, 2014; Coey, 2015; Barbaresco *et al*., 2015; Wittman, 2015) and the Children's Health Insurance Programme (Gresenz *et al*., 2013; Goldstein *et al*., 2014; Guevara *et al*., 2014).

In France, several studies have examined the effect of CHI coverage on the use of healthcare (Mormiche, 1993; Caussat and Glaude, 1993; Genier, 1998; Chiappori *et al*., 1998; Buchmueller *et al*., 2004), while fewer studies have analysed the adoption and impact of means‐tested health insurance programmes (Grignon *et al*., 2008; Guthmuller and Wittwer, 2012; Guthmuller *et al*., 2014). To the best of our knowledge, the paper of Grignon *et al*. (2008) was the first to focus on the causal impact of CMUC on healthcare utilisation. More specifically, the authors analysed the effect of being a CMUC recipient on healthcare utilisation by differentiating three types of transition to CMUC at the time of its introduction, including former recipients of the means‐tested assistance plan called Aide Médicale Générale for whom the transition to CMUC was automatic. The authors found no significant effect on utilisation for individuals who were previously covered by Aide Médicale Générale but a significant impact for people who enrolled voluntarily, especially for those not previously covered by any CHI. These individuals are more likely to use and spend more on generalist care and specialist care, as well as prescription drugs. The 2008 study of Grignon *et al*. investigated the effects of CMUC on healthcare expenditures within the CMUC eligible population at the time of its introduction.

In contrast, this study seeks to examine the impact of the CMUC eligibility threshold on the healthcare use of poor individuals with an income around the eligibility threshold. More precisely, our aim is to see whether, all other things held constant, individuals with an income just above the eligibility cut‐off point use less healthcare than individuals eligible for the plan. If we observe this difference, we could then conclude that the chosen income cut‐off point introduces a gap or a ‘threshold effect’ of the CMUC plan between both groups of individuals. Thus, we use this approach to assess the efficacy of the CMUC plan and the ACS programme in improving equity in access to healthcare in France.

Thanks to information on the selection rule to qualify for the plan, we were able to assess CMUC eligibility and adopt a regression discontinuity approach. Regression discontinuity designs can be viewed as local randomised experiments. This approach, first implemented by Thistlethwaite and Campbell in 1960 to analyse the impact of merit awards on academic outcomes, is increasingly being used to evaluate the effect of programmes in non‐experimental settings; for a comprehensive literature review, see Lee and Lemieux (2010). Several recent papers investigate the effect of health insurance on health and healthcare utilisation using a regression discontinuity design. In 2010, Hullegie and Klein disentangle the effect of being privately insured in Germany on self‐assessed health, the number of nights spent in hospitals and the number of doctor visits, using income as a running variable. Some papers estimate the effect of Medicare coverage on mortality and healthcare utilisation using age as an assignment variable (Card *et al*., 2008; Card *et al*., 2009). Other studies analyse the effect of Medicaid on health and health consumption using age or birth date and family income as a forcing variable (Card and Shore‐Sheppard, 2004; De la Mata, 2012; Dague, 2014; Coey, 2015).

In this paper, healthcare use is measured according to the number of doctor visits. Doctor visits are in fact a good indicator of healthcare inequalities and access to primary care in France (Bourgueil *et al*., 2012). The impact of CMUC eligibility is then estimated based on the total number of visits to the doctor, the number of general practitioner (GP) visits and the number of specialist visits.

Our sample consists of 2232 low‐income individuals enrolled in the Health Insurance Fund (Caisse Primaire d'Assurance Maladie, CPAM) and recipients of social benefits from the Family Allowance Fund (Caisse d'Allocations Familiales, CAF) in an urban area in Northern France. These individuals have an income per consumption unit (CU) of up to 30% above or below the CMUC eligibility cut‐off point. Individuals below this cut‐off point can benefit from the CMUC plan, and individuals above this cut‐off point are eligible for ACS. Our data set includes claim data from all doctor visits for the years 2008 and 2009 as well as information on 2007 and 2008 resources.

In total, we find no significant impact of the CMUC eligibility threshold on the number of doctor visits, neither on GP visits nor on specialist visits. But this absence of effect in the full population does not mean the CMUC cut‐off point has no effect on healthcare utilisation at all. The eligibility threshold may actually have an impact in certain subgroups of the population. Among the subsample of adults under 30 years old, for instance, we find that eligible individuals are more likely to see a specialist and have, on average, significantly more specialist visits than non‐eligible individuals. This specific impact of the CMUC cut‐off point among young adults may be explained by the fact that young adults are less likely to be covered by a CHI plan when they are not recipients of the CMUC plan.

The remainder of this paper is organised as follows. Section 2 describes the institutional background, and the data and the estimation strategy are explained in Section 3. Section 4 presents the estimation results regarding the impact of the free plan on the number of doctor visits and the robustness checks. Our findings are discussed in Section 5.

## 2 Institutional Background

### 2.1 The French health insurance system

The National Health Insurance fund provides public, compulsory and universal health insurance that covers three‐quarters of all overall healthcare expenses (76% in 2013 according to French National Health Accounts (Zaidman *et al*., 2014)). Patients generally pay approximately 50% of costs for routine care 1Except for patients suffering from a long‐term illness, healthcare expenses for chronic diseases are 100% covered. (Le Bras and Tabuteau, 2012). The fund is particularly important for certain types of care, such as consultations with extra fees, medications, glasses and other types of prostheses and dental care.

In addition, individuals can purchase a CHI plan upon the payment of variable premiums to cover any remaining copayments. In France, CHI is not only complementary to NHI, as CHI covers copayments, but also supplements NHI as it can reimburse charges that exceed the statutory fee or healthcare expenses that are not covered at all by NHI (for instance, excess fees for doctor visits, non‐reimbursed medication and private hospital rooms). This system raises the issue of financial access to care and the affordability of CHI for the poorest populations. To improve access to CHI for the poorest, two programmes exist in France: the CMUC programme, which is a free CHI plan available to the poorest households in France (Grignon *et al*., 2008), and the ACS voucher programme for poor households whose income is slightly above the CMUC threshold. 2In January 2009, households with an income level between the CMUC threshold and 20% above it were eligible for ACS. This programme provides financial incentives for uninsured households to acquire a CHI plan. It also partially reimburses those who already purchased a policy and gives them an incentive to purchase a better‐quality CHI plan. Studies have shown, however, that take‐up of this financial incentive remains low (Guthmuller *et al*., 2014).


### 2.2 The Couverture Maladie Universelle Complémentaire plan

Couverture Maladie Universelle Complémentaire was introduced in France in 2000 and provides access to free CHI coverage for the bottom 10% of households. In 2007, households earning less than 7272 Euros per year for a single person in metropolitan France were eligible. This cut‐off was 7447 Euros in 2008 (Fonds CMU, 2011a) and is dependent on the number of family members. Eligibility is calculated based on the 12 months of family income earned prior to applying. The eligibility threshold has been revaluated each year since the plan's introduction in 2000 and has been set at 8645 Euros since 2014. The elderly (i.e. those aged 60 or above) and the disabled are not eligible for the free plan because the minimum income they receive from the government is higher than the cut‐off. All resources including family allowances and housing benefits are taken into account. 3A detailed description of resources taken into account for the eligibility assessment can be found in the Social Security Code (Code de la Sécurité Sociale, 2011 and Fonds CMU, 2011b). In October 2014, 4 555 795 individuals received this free plan (Fonds CMU, 2015a). In practice, eligibility assessments are performed by local offices of the national health insurance fund (CPAM); CMUC can be directly taken out by the CPAM or by a CHI provider, and the application must be renewed annually.

The offered coverage is equivalent to a ‘medium‐quality’ CHI plan. Patients' contributions are covered (fees, hospital co‐payments, etc.), and they have free access to care at the point of use. 4Overall, access to doctors in France is regulated by financial constraints. Patients must pay a fixed portion of the regulated consultation fee (30%) at the point of use. Access to specialist care is regulated by the preferred doctor scheme in which patients register a family doctor, usually a GP, and are covered by standard rates of the NHI when seeing a specialist after being referred by the family doctor. Direct access to specialist care and to another GP is still possible, but patients must pay a higher co‐payment (70% instead of 30% within the healthcare pathway). 85% of individuals had registered a family doctor in 2008 (Assurance Maladie, 2009 2009). Physicians must treat patients covered by CMUC who are willing to be treated and cannot overbill them even though they overbill their other patients (excess fees for visits, dentures or optical care). 5In France, healthcare fees are agreed upon by the national health insurance system, and reimbursement is based on these statutory fees. Some doctors (those belonging to the unregulated payment sector), however, have the right to set prices exceeding this statutory fee for their visits or for certain types of care. This was the case for 24% of all physicians in 2010. This rate is 41% on average for specialist care but varies by specialty. For instance, 85% of surgeons have the right to ask for higher fees versus 32% for paediatricians (CNAMTS, 2011 2001). Healthcare packages beyond the base reimbursement for certain services (such as optical and dental care) are covered. The level of coverage varies greatly among CHI plans; the best plans cover the full price (including excess fees) for specialist visits, while the baseline plans cover the statutory co‐payment only (Garnero and Le Palud, 2013). Among the low‐income population and especially those eligible for the ACS voucher, individuals covered by a CHI plan are usually covered by the base plan (Fonds CMU, 2015b).

## 3 Data and Methods

### 3.1 Data and sample selection

Our sample of individuals consists of poor recipients of family benefits from the CAF and the CPAM of Lille, an urban area in Northern France. These individuals were identified at the end of 2008 on the basis of their 2007 tax‐declared incomes as potentially eligible either for the CMUC plan or for the ACS voucher programme 6The primary objective of this data extraction was to provide a sample of all individuals eligible for the ACS voucher in 2009 in the Lille metropolitan area as part of a randomised experiment to assess the effect of the amount of the voucher on take‐up (Guthmuller *et al*., 2014). Given the timeframe to complete the experiment, however, only 2007 tax‐based income was observed. Thus, the extraction includes individuals identified as eligible to ACS for both 2009 and 2008. Consequently, this sample consisted of individuals eligible for ACS as well as individuals eligible for CMUC, often with an income near the threshold, because of the imperfect observation of eligibility with the information available in 2009. It is precisely this characteristic of the sample that we use in this paper to identify the effect of CMUC on doctor visits. (Guthmuller *et al*., 2014). This programme is open to poor households whose income were up to 20% above the CMUC cut‐off in 2009. Since January 2013, the eligible population has been extended to individuals with an income up to 35% above the CMUC cut‐off. Our sample consists of individuals eligible for ACS and individuals eligible for CMUC, often with an income near the threshold.

Based on this sample and on updated information on reported income taxes and any monthly social benefits in 2007 and 2008, we accurately retrieved the annual income per CU used to assess eligibility for CMUC. 7A detailed description of the income calculation is available upon request. We then retained for the years 2008 and 2009 those individuals whose household income per CU one year earlier was within a range of + or ‐ 30% around the CMUC eligibility threshold. We excluded from our sample individuals who benefit from a disability allowance or are recorded by the CPAM as being fully covered for a chronic disease, 8In a previous analysis, we did not exclude individuals with chronic diseases to study the causal effect of the CMUC plan using eligibility as an instrumental variable. As the effects we found are mainly driven by these individuals, we excluded them of the sample in the present study (Guthmuller and Wittwer, 2012). in addition to individuals over the age of 60; as previously indicated, these people are generally not eligible for CMUC. 9From the 4209 individuals initially identified by the CAF (Guthmuller *et al*., 2014), we excluded individuals older than 60 (18% of the sample). We also excluded 28% who had a chronic disease or a disability and 27% who had an income above or below the income range of + or −30% around the CMUC eligibility threshold.


For each individual belonging to this sample and for each year, we collected data from the CAF and the CPAM. The Family Allowances Fund provided information on family characteristics for the years 2007 and 2008, the number of members in the household and the family composition (couple, single, a couple with children, single with children), as well as age, gender and employment status. The CPAM provided administrative data on the number of doctor visits, the number of GP visits and the number of specialist visits. This database also includes monthly information on the CHI coverage status and whether the individual is 100% covered for a chronic disease.

Our sample consists of 2232 observations (1221 in 2008 and 1011 in 2009). We define individuals as CMUC recipients if they are covered by the CMUC plan for at least the first 6 months of the year. As eligibility is assessed on income in the 12 months prior to application, it is possible that individuals who appear to be ineligible for CMUC on 31 December are still considered CMUC recipients the following year, because they could be eligible 6 months earlier or 6 months later. Battistin *et al*. (2009) showed that if we assume this measurement error of eligibility orthogonal to treatment and outcome variables and under the assumption that we observe the true eligible income for part of the population, the estimation strategy we follow in this paper remains valid. 10This assumption is explained in more detail in the online appendix.


Under this definition, 8% of individuals are benefiting from the free plan, and given the information on family income per CU, 29% of individuals are eligible for it. This means that within the CMUC‐eligible population, only 16% are covered by the free plan, while 55% are covered by a CHI plan. 11An individual covered for at least 6 months in a year by a CHI plan was considered as benefiting from a CHI plan for the entire year. This rate is quite low, far below the estimated take‐up rate in the entire eligible population (84%; Fonds CMU, 2012). One must keep in mind that individuals in our sample have resources close to the threshold; in this view, uncertainty about eligibility for the programme could explain the low take‐up rate (Remler *et al*., 2001; Currie, 2006).

### 3.2 Estimation strategy

The aim of this study is to analyse the effect of the CMUC eligibility threshold on doctor visits. Our identification strategy relies on the assignment variable *Z*_*it* − 1_, ‘household income’, which is defined as the household income used for the CMUC eligibility assessment of individual *i* at *t* ‐ 1, 1 year before CMUC application. Individual *i* can only qualify for CMUC if his/her household income is below the CMUC cut‐off, *c*_*it* − 1_. We define the dummy variable indicating whether individual *i* is eligible for CMUC at time *t* as *eliCMUC*_*it*_ = *I*(*Z*_*it* − 1_ < *c*_*it* − 1_). *EliCMUC*_*it*_ ≠ *CMUC*_*it*_.

We then adopt a regression discontinuity approach thanks to the selection rule to qualify for CMUC. This approach relies on three assumptions. First, individuals do not manipulate their income in order to become eligible for the free plan, so being CMUC‐eligible is a random status for individuals with income around the threshold. As we cannot definitely exclude this possibility, we discuss and check the existence of income manipulation in Section 4.4. In particular, we perform several tests to validate this identification assumption. Second, the assignment variable has a discontinuous impact on the probability of being a CMUC recipient. Because we observe CMUC eligibility with good precision, we expect a discontinuous impact of the eligibility threshold. We perform regressions in the following section to check the validity of this second assumption. Third, there is a monotonic relationship between CMUC coverage and eligibility around the threshold. This last assumption is given by construction, as individuals with income above the threshold cannot benefit from the free plan (Battistin and Rettore, 2008).

Therefore, to estimate the effect of CMUC eligibility, ideally, we would non‐parametrically compare the outcome variable of interest for people below and above the threshold in a small interval. 12Formally and as shown by Hahn *et al* (2001); Imbens and Lemieux (2008); and Lee and Lemieux (2010), the effect of CMUC eligibility *δ* can be estimated without bias on outcome variable *y* as: $\delta =\underset{z\to c^{-}}{\text{lim}}E\left[Y/Z=z\right]-\underset{z\to c^{+}}{\text{lim}}E\left[Y/Z=z\right]$. As the income bandwidth of our sample is relatively large, we assess (i) whether income has a significant influence on healthcare utilisation outcomes (independently of the eligibility effect) and (ii) whether individual characteristics are the same in a statistical sense in eligible and non‐eligible populations. Furthermore, our sample is too small to allow us to narrow the household income bandwidth.

We then follow the parametric strategy introduced by van der Klaauw in 2002. We first check the validity of the second and third assumptions by estimating the likelihood of being a CMUC recipient using a linear probability model as follows:

(1) $$E\left[CMUC_{\text{it}}|X_{\text{it}},\text{eliCMU}C_{\text{it}},Z_{\text{it}-1}\right]=\alpha +\beta *X_{\text{it}}+\delta *\text{eliCMU}C_{\text{it}}+\;\text{eliCMU}C_{\text{it}}*k_{g1}\left(Z_{\text{it}-1}\right)+\left(1-\text{eliCMU}C_{\text{it}}\right)*k_{g2}\left(Z_{\text{it}-1}\right)+E\left[u_{\text{it}}|X_{\text{it}},\text{eliCMU}C_{\text{it}},Z_{\text{it}-1}\right]$$

where *CMUC*_*it*_(=0,1) is a variable indicating whether *i* is covered by the free plan in year *t* and EliCMUC_it_ = *I*(*Z*_*it* − 1_ < *c*_*t* − 1_). *δ* measures the effect of CMUC eligibility, that is the height of the discontinuity around the threshold. We include polynomial functions of household income *k*_*g*1_ (*Z*_*it* − 1_) and *k*_*g*2_ (*Z*_*it* − 1_) in Equation (1) to capture the (continuous) effect of income on the probability of being a CMUC recipient. In the same way, we add other covariates, *X*_*it*_, in order to control for differences between CMUC eligible and CMUC non‐eligible individuals. *u*_*it*_ is the unobserved error component with *E*(*u*_*it*_|*X*_*it*_, *eliCMUC*_*it*_, *Z*_*it* − 1_) = 0.

We are specifically interested in two outcomes: the likelihood of seeing a doctor and the number of doctor visits.

#### Likelihood of seeing a doctor

We estimate the impact of eligibility on the first outcome using a logistic model with the following equation:

(2) $$E\left[V_{\text{it}}|X_{\text{it}},\text{eliCMU}C_{\text{it}},Z_{\text{it}-1}\right]=\exp[a+b*X_{\text{it}}+d*\text{eliCMU}C_{\text{it}}+\text{eliCMU}C_{\text{it}}*h_{g1}\left(Z_{\text{it}-1}\right)+\left(1,-,\text{eliCMU},C_{\text{it}}\right)*h_{g2}\left(Z_{\text{it}-1}\right)+E\left(w_{\text{it}}|X_{\text{it}},\text{eliCMU}C_{\text{it}},Z_{\text{it}-1}\right)]$$

where *V*_*it*_(=0,1) is a binary variable indicating if individual *i* visited a doctor at least once during year *t* and *eliCMUC*_*it*_ = *I*(*Z*_*it* − 1_ < *c*_*t* − 1_). *d* measures the impact of being CMUC eligible. As in Equation (1), we add polynomial functions of household income, *h*_*g*1_ (*Z*_*it* − 1_) and *h*_*g*2_ (*Z*_*it* − 1_) and *X*_*it*_ a matrix of control variables. *w*_*it*_ is the unobserved error component with *E*(*w*_*it*_|*X*_*it*_, *eliCMUC*_*it*_, *Z*_*it* − 1_) = 0.

#### Number of doctor visits

We estimate the effect of CMUC eligibility on the number of doctor visits using a count data model specified as follows:

$$E[N_{\text{it}}|X_{\text{it}},\text{eliCMU}C_{\text{it}},Z_{\text{it}-1}]=\exp[[A+B*X_{\text{it}}+D*\text{eliCMU}C_{\text{it}}+\;\text{eliCMU}C_{\text{it}}*H_{g1}\left(Z_{\text{it}-1}\right)+\left(1-\text{eliCMU}C_{\text{it}}\right)*H_{g2}\left(Z_{\text{it}-1}\right)+E\left(W_{\text{it}}|X_{\text{it}},\text{eliCMU}C_{\text{it}},Z_{\text{it}-1}\right)]\;\left(2'\right)$$

where *N*_*it*_ is the number of visits of *i* in year *t* and *eliCMUC*_*it*_ = *I*(*Z*_*it* − 1_ < *c*_*t* − 1_). *D* is our parameter of interest. As in Equation (2), we add polynomial functions of household income, *H*_*g*1_(*Z*_*it* − 1_) and *H*_*g*2_(*Z*_*it* − 1_) and *X*_*it*_ a matrix of control variables. *w*_*it*_ is the unobserved error component with *E*(*W*_*it*_|*X*_*it*_, *eliCMUC*_*it*_, *Z*_*it*_) = 0.

Because the number of doctor visits is over‐dispersed, we use a negative binomial II model to estimate *D*. This is also the model that best fits our data. 13To estimate the impact of CMUC eligibility on doctor visits, we use two types of models. A negative binomial II model is applied to estimate the number of visits, and a zero‐truncated negative binomial II model is used to find the conditional number of visits. We choose to use a negative binomial II specification over a Poisson specification to explicitly account for the over‐dispersion feature of the data. We also test the necessity of using of zero‐inflated over a standard negative binomial model and find no significant improvement. Vuong's closeness test does not significantly reject that both models were equally close to the observed distribution of the data in all estimates. The model selection analysis is available upon request.


We observe the assignment variable to CMUC (household income per CU) in 2007 (2008) and estimate the effect on outcome variables in 2008 (2009). Estimates are run on the pooled dataset 14We checked for the necessity of including individual fixed effects in the model, and it appeared that the pooled estimation was the best specification. Pooled estimations were run with cluster‐robust standard errors at the individual level. and with different polynomial functions of income to check the robustness of our results. The baseline specification includes a linear function of income per CU and a year dummy, individual and family characteristics.

## 4 Results

### 4.1 Characteristics of the sample

Table 1 reports descriptive statistics on doctor visits. CMUC‐eligible individuals show a greater number of visits on average compared with non‐eligible individuals: 4.0 visits versus 3.6 visits, respectively. The *p*‐value of the difference test in the mean is 0.0036. Specifically, CMUC‐eligible individuals visit GPs and specialists significantly more often (*p*‐values equal to 0.0160 and 0.0054, respectively). The proportion of individuals with at least one doctor or one GP visit is similar across eligible and ineligible individuals. In contrast, this proportion is higher for specialist visits: 39.9% among the eligible population and 36.0% among those not eligible for the plan, with the *p*‐value of the difference test in proportion being 0.0409.

**Table 1:** Outcome variables among the eligible population (per year, pooled data, 2008, 2009)

N = 2232	Doctor visits (Total)		GP visits		Specialist visits	
CMUC	Eligible	Non‐eligible	Eligible	Non‐eligible	Eligible	Non‐eligible
*N*	639	1593	639	1593	639	1593
At least one visit (%)	83.7	82.4	80.3	78.9	39.9	36.0
Number of visits	4.0	3.6	3.2	2.9	0.8	0.7
Cond. number of visits	4.8	4.3	3.9	3.6	2.1	1.9
*N*	*535*	*1312*	*513*	*1257*	*255*	*573*

Notes: Doctor visits (total, GP, specialist) are those observed in 2008 and 2009.

CMUC, Couverture Maladie Universelle Complémentaire; GP, general practitioner.

Regarding their individual and family characteristics, the average age of the sample is 37 years. There are slightly more women, younger individuals, singles with children and unemployed individuals among CMUC‐eligible individuals, as compared with fewer couples with children and employed individuals within the non‐eligible population. These differences are not statistically significant when controlling for income (Table 2).

**Table 2:** Individual characteristics (pooled, 2007, 2008)

CMUC	Eligible	Non‐eligible	Δ without controls (a)	Δ with controls (b)
Demographic characteristic				
Age	37.0	36.6	0.4	0.5
Female	57.6%	48.3%	0.093***	0.034
Employment and family characteristics				
Employed	39.6%	54.6%	‐0.150***	0.016
Family with children	75.9%	86.4%	‐0.105***	‐0.017
N	639	1593		

Notes: CMUC, Couverture Maladie Universelle Complémentaire.

Column (a) reports the difference in mean between the eligible group and the non‐eligible group and the statistical significance of the difference. The difference in mean after controlling for income and a year dummy for 2008 is displayed in column (b). Statistical significance levels are

* *p*<=10%;

** *p*<=5%;

*** *p*<=1%.

### 4.2 Couverture Maladie Universelle Complémentaire participation around the threshold

**Figure 1:**
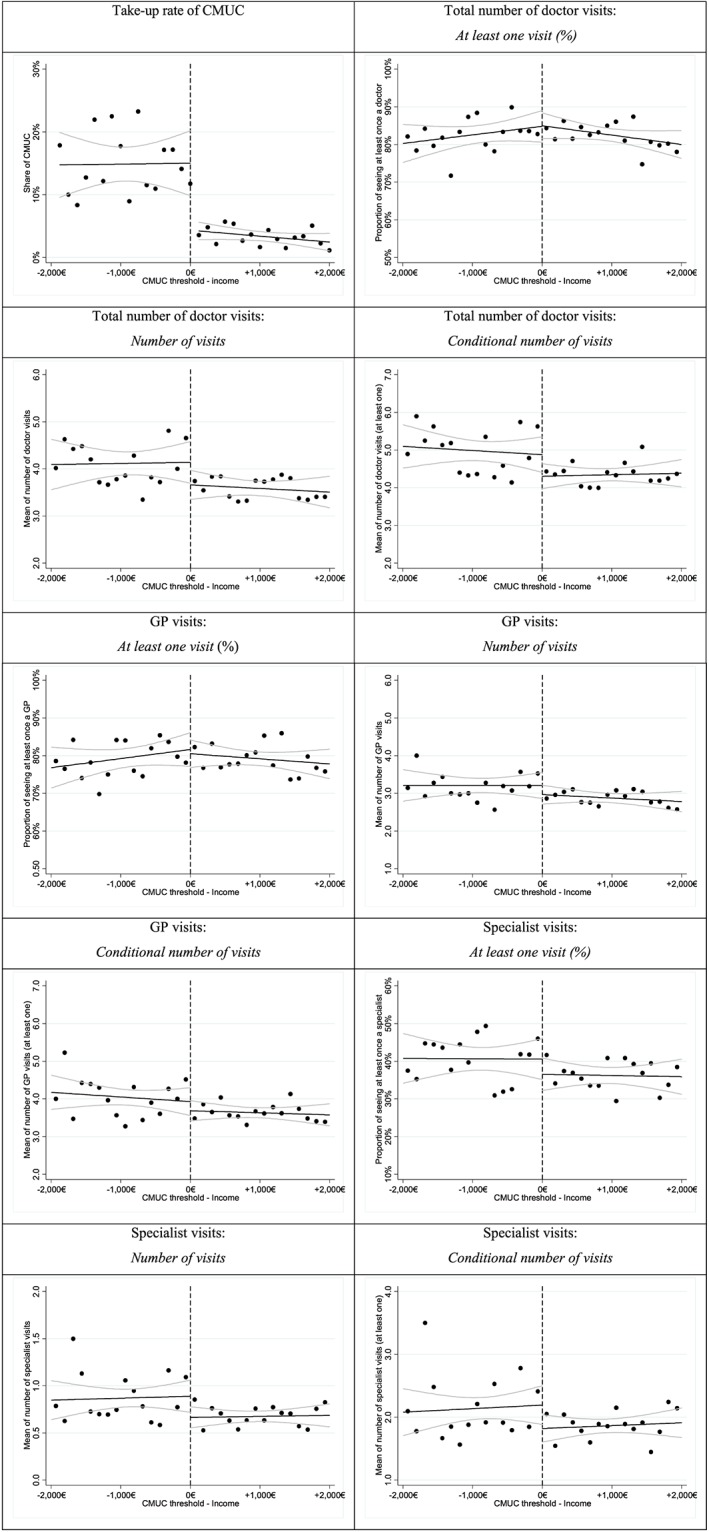
Discontinuity in the number of doctor visits around the cut‐off point (Pooled data, bin size = 125 Euros). *Notes*: As the eligibility threshold differed in 2007 and 2008, income per consumption unit (CU) was normalised by subtracting the corresponding eligibility cut‐off point. A negative value indicated that income was below the threshold and the individual was eligible for Couverture Maladie Universelle Complémentaire (CMUC). Our sample consisted of individuals with a normalised income per CU between ±2000 Euros around the eligibility threshold. [Colour figure can be viewed at wileyonlinelibrary.com]

We examine whether there is a discontinuity in the probability of being a CMUC recipient over the assignment variable (income) around the threshold. Figure 1 shows the proportion of persons covered by CMUC over the income per CU. Each dot is the percentage of CMUC recipients in each income per CU bin of 125 Euros wide and the solid line is the corresponding linear smooth plot with 95% confidence level curves. We observe a clear discontinuity of the proportion of CMUC recipients around the CMUC eligibility cut‐off point (normalised to zero).

The proportion of CMUC recipients among the eligible population is 15.6% on average, and, as previously discussed, because of eligibility measurement errors, 3.6% of CMUC non‐eligible individuals were actually covered by the free plan.

This discontinuity is confirmed by estimates of the probability of CMUC participation as specified in Equation (1), (Table 3). The impact of eligibility (measured by the dummy variable *EliCMUC*) on CMUC participation is significant whatever the polynomial function chosen, and controlling for individuals and family characteristics does not modify the estimates.

**Table 3:** Discontinuity in the probability of CMUC participation

N=2232	Without controls (1)	With Controls (2)
	*Without running variable*	
EliCMUC	0.118*** (0.0116)	0.114*** (0.0119)
	*Linear specification of the running variable (same coefficient at both sides of the threshold)*	
EliCMUC	0.103*** (0.0315)	0.101*** (0.0312)
	*Linear specification of the running variable (different coefficients at both sides of the threshold)*	
EliCMUC	0.108*** (0.0380)	0.107*** (0.0378)
	*Quadratic specification of the running variable (same coefficient at both sides of the threshold)*	
EliCMUC	0.107*** (0.0381)	0.107*** (0.0379)

*Notes:* (1) All regressions are linear probability models including a year dummy for 2008 and the displayed specification of income. (2) All regressions are linear probability models including a year dummy for 2008, individual and family characteristics in 2008 and the displayed specification of income. Robust standard errors are shown in parentheses. Statistical significance levels:

* p<=10%;

** p<=5%;

*** p<=1%.

### 4.3 Impact of CMUC on doctor visits

Figure 1 also reports the use of doctor visits around the CMUC cut‐off point over the income per CU. Each dot is either the proportion of having seen a doctor at least once in a year, the number of visits or the conditional number of visits per year in each income per CU bin of 125 Euros wide. The solid line is the corresponding linear smooth plot with 95% confidence level curves. Graphs are displayed for total doctor visits, GP visits and specialist visits.

These graphs provide additional information on the distribution of doctor visits around the CMUC threshold. If the number of visits is higher on average and matches the descriptive statistics in Table 1, the differences appear to be less clear over the income distribution.

This result is confirmed by the estimates of the number of visits around the CMUC cut‐off point. When controlling for income and individual and family characteristics, the estimates of the differences in the number of visits in Table 4 are not statistically significant. Estimates are conducted with and without individual and family characteristics in addition to income variables. 15The robustness of these results is tested for different polynomial functions of income and is presented in the online appendix file.


**Table 4:** Impact of CMUC eligibility on doctor visits

Full sample	Without controls (1)			With controls (2)		
Bandwidth	Doctor visits (total)	GP visits	Specialist visits	Doctor visits (total)	GP visits	Specialist visits
At least one visit (a)						
OR	0.945	1.113	1.168	0.977	1.144	1.141
(s.e.)	(0.2878)	(0.3245)	(0.2772)	(0.3051)	(0.3399)	(0.2762)
Number of visits (b)						
IRR	1.003	0.954	1.202	1.010	0.972	1.159
(s.e.)	(0.1103)	(0.1054)	(0.2436)	(0.1101)	(0.1068)	(0.2322)
N	2232	2232	2232	2232	2232	2232
Cond. number of visits (b)						
IRR	1.011	0.932	1.074	1.022	0.952	1.084
(s.e.)	(0.0976)	(0.0868)	(0.1492)	(0.0976)	(0.0882)	(0.1510)
N	1847	1770	828	1847	1770	828

Notes: CMUC, Couverture Maladie Universelle Complémentaire; GP, general practitioner.

Notes: (a) All regressions are logit models. Odds ratios (OR) are reported. (b) All regressions are negative binomial models; the exponential of the estimated coefficient is reported, incident rate ratio, IRR. (1) Regressions include family income and a year dummy for 2008. (2) Regressions include family income, a year dummy for 2008, individual and family characteristics. Robust standard errors (s.e.) are shown in parentheses. Statistical significance levels: *p ≤ 10%; **p ≤ 5%; ***p ≤ 1%.

Part of the explanation for this absence of differences may be found in the proportion of CHI coverage around the CMUC threshold. The proportion of individuals without any CHI coverage (either CMUC or CHI coverage) is statistically similar between the eligible population and the non‐eligible population in our sample (Table 5, 29.7% vs. 27.8%). In fact, the remaining non‐recipients of the CMUC plan among the eligible population were covered by a CHI plan. Further, this proportion is not statistically different from the proportion of enrolees in a CHI plan among the non‐eligible group after controlling for individual and family characteristics. Recall that the majority of our sample is employed, and individuals may be covered by a CHI plan through their employers. This might not be the case for younger individuals, however, who have less ready access to more stable employment and employers' CHI plans.

**Table 5:** Proportion of CHI coverage around the CMUC threshold

CMUC	Eligible	Non‐eligible	Δ without controls (a)	Δ with controls (b)
Complementary health insurance				
CMUC	15.3%	3.6%	0.117***	0.103***
Complementary health insurance	54.9%	68.5%	‐0.136***	‐0.088
No coverage	29.7%	27.8%	‐0.019	‐0.015
N	639	1593		

Notes: CHI, complementary health insurance; CMUC, Couverture Maladie Universelle Complémentaire.

Column (a) reports the difference in mean between the eligible group and the non‐eligible group and the statistical significance of the difference. The difference in mean after controlling for income and a year dummy for 2008 is displayed in column (b). Statistical significance levels are

* *p*<=10%;

** *p*<=5%;

*** *p*<=1%.

### 4.4 Impact in the younger population

**Figure 2:**
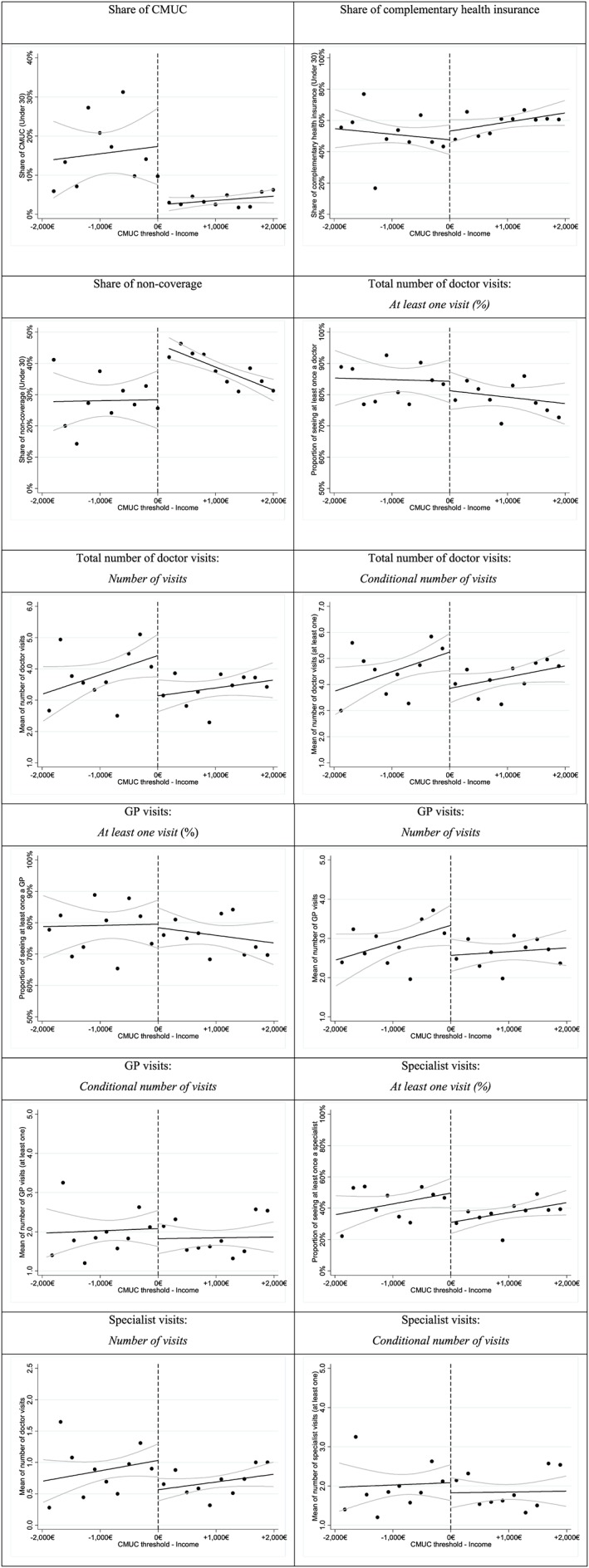
Discontinuity in the take‐up rate of CMUC, in the share of CHI plans, in the proportion of non‐coverage, and in the number of doctor visits around the cut‐off point amongst the younger adults. *Notes*: As the eligibility threshold differed in 2007 and 2008, income per consumption unit (CU) was normalised by subtracting the corresponding eligibility cut‐off point. A negative value indicates that income was below the threshold and the individual was eligible for Couverture Maladie Universelle Complémentaire (CMUC). Our sample consists of individuals under 30 years old with a normalised income per CU between ±2000 Euros around the eligibility threshold.

To investigate this result further, we examine the proportion of CHI coverage around the CMUC threshold among the subsample of individuals aged under 30 years. 16We ran the analysis for different age groups, and the results were only statistically different among the subsample of adults under 30 years old. We also ran the analysis among the subsample of 30 years old and older. The results of this analysis are available upon request. Table 6 reports descriptive statistics of the younger adult population between the eligible group and the non‐eligible group. Similarly to the full sample, the proportion of CMUC recipients is 16.8% among the eligible group and 4.1% among the non‐eligible group. The difference on average between the two groups is statistically significant. Correspondingly, Figure 2 reports a discontinuity in the proportion of CMUC coverage around the CMUC cut‐off point over the income per CU amongst the younger adults. On the contrary, the proportion of individuals with no coverage (neither the CMUC nor CHI plan) among potential CMUC beneficiaries is significantly lower than in the non‐eligible group; the proportion was 28.1% versus 37.0%, respectively (Figure 2, Table 6). Once we control for income, the difference remains statistically significant. Regarding their demographic characteristics, young adults are similar across the two subgroups (Table 6).

**Table 6:** Individual characteristics around the CMUC threshold among younger adults

CMUC Eligibility	Eligible	Non‐eligible	Δ without controls (a)	Δ with controls (b)
Complementary health insurance				
CMUC	16.8%	4.1%	0.127***	0.152**
Complementary health insurance	55.1%	58.9%	‐0.038	‐0.046
No coverage	28.1%	37.0%	‐0.088**	‐0.0978*
Demographic characteristics				
Age	25.2	25.3	‐0.086	‐0.380
Female	65.3%	57.0%	0.082**	0.0363
Employed	47.3%	52.0%	‐0.047	‐0.046
Family with children	71.8%	74.8%	‐0.029	‐0.027
N	167	365		

Notes: CMUC, Couverture Maladie Universelle Complémentaire.

Column (a) reports the difference in mean between the eligible group and the non‐eligible group and the statistical significance of the difference. The difference in mean after controlling for income and a year dummy for 2008 is displayed in column (b). Statistical significance levels are

* *p*<=10%;

** *p*<=5%;

*** *p*<=1%.

Figure 2 reports the use of doctor visits around the CMUC cut‐off point over the income per CU amongst the younger adults. The average number of doctor visits is slightly higher within the eligible population, but the difference is not significant apart from the number of visits to a specialist. This difference is mainly driven by differences in access to specialists. The proportion of individuals seeing a doctor or specialist at least once is significantly higher in the CMUC‐eligible population (Figure 2). In effect, among the younger adults, eligible individuals visit, on average, 1.5 more doctors and are 3.2 times more likely to see a specialist than the non‐eligible individuals 17Estimates were conducted with and without individual characteristics in addition to income. The robustness of these results was tested for different polynomial functions of income and is presented in the online appendix. (Table 7).

**Table 7:** Impact of CMUC eligibility on doctor visits among younger adults

Under 30	Without controls (1)			With controls (2)		
Bandwidth	Doctor visits (total)	GP visits	Specialist visits	Doctor visits (total)	GP visits	Specialist visits
At least one visit (a)						
OR	1.265	1.214	2.996**	1.380	1.310	3.212**
(s.e.)	(0.7910)	(0.7171)	(1.4074)	(0.9077)	(0.8026)	(1.5531)
Number of visits (b)						
IRR	1.599**	1.344	3.058***	1.584**	1.323	3.247***
(s.e.)	(0.3438)	(0.2998)	(1.1705)	(0.3422)	(0.2974)	(1.2162)
N	532	532	532	532	532	532
Cond. number of visits (b)						
IRR	1.514**	1.268	1.550	1.503**	1.245	1.564
(s.e.)	(0.2834)	(0.2359)	(0.4365)	(0.2761)	(0.2295)	(0.4336)
N	429	409	210	429	409	210

Notes: CMUC, Couverture Maladie Universelle Complémentaire; GP, general practitioner.

(a) All regressions are logit models; odds ratio (OR) are reported. (b) All regressions are negative binomial models; the exponential of the estimated coefficient is reported, incident rate ratio, IRR. (1) Regressions include family income and a year dummy for 2008. (2) Regressions include family income, a year dummy for 2008, individual and family characteristics. Robust standard errors (s.e.) are shown in parentheses. Statistical significance levels:

* *p* ≤ 10%;

** *p* ≤ 5%;

*** *p* ≤ 1%.

### 4.5 Robustness checks

To validate the identification assumptions underlying the regression discontinuity analysis, we perform several tests. 18Results of these tests are detailed in the online appendix.


We first check the absence of manipulation of the selection variable around the eligibility threshold. As mentioned previously, it is conceivable that some individuals would be tempted to work less to become or remain eligible for CMUC. However, amongst our sample of low‐income individuals, when one has an employment it is within a salaried work contract for which they have a limited degree of negotiation over their salary. Individuals are also not easily able to adjust the number of hours worked to remain eligible. For this reason and considering the narrow income bandwidth and the difficulty of anticipating CMUC eligibility, it appears unlikely that a significant proportion of the eligible beneficiary households manipulated their income to receive the free plan. To test this hypothesis, we draw the distribution of income per CU around the eligibility threshold and check whether there is an accumulation of observations just below the threshold. The presence of a discontinuity in the density function at the cut‐off point is tested and rejected using a two‐step test proposed by McCrary (2008). 19The results of these tests, presented in the online appendix, confirm the absence of discontinuity in the density of the assignment variable.


In addition to the discontinuity in the probability of being a CMUC recipient over the assignment variable around the threshold, we verify the absence of any other discontinuity in the likelihood of being a CMUC recipient at each side of the eligibility threshold. 20See additional material in the online appendix.


## 5 Discussion

In this study, we focused on the impact of the CMUC eligibility threshold on doctor visits within a population with an income around the CMUC cut‐off point. Using the means‐tested feature of the selection rule to qualify for the free plan, we adopted a regression discontinuity design.

Within the full sample, we found no significant impact of the CMUC eligibility threshold on the number of doctor visits, neither on GP visits nor on specialist visits. The similar proportion of CHI coverage rate amongst the observed population on both sides of the CMUC threshold might explain this absence of difference in doctor visits between eligible and the non‐eligible individuals.

However, one must be careful in interpreting this lack of impact of eligibility for the CMUC plan as the result of an efficient threshold choice, because the eligibility cut‐off point does not lead to less access to healthcare for the population above the threshold. Individuals with income above the eligibility threshold must carry the financial burden of being covered by a CHI plan that is generally of lower quality compared with the CMUC plan.

In addition, this absence of effect in the full population does not mean the CMUC cut‐off point has no effect on healthcare utilisation at all. Actually, when we focused the analysis on young adults, a different picture emerged. Among this population, CMUC eligibility showed a significant impact on doctor visits and, in particular, on the probability of seeing a specialist; eligible individuals were more likely to see a specialist and had, on average, significantly more specialist visits than non‐eligible individuals. This result may be due to the fact that young adults are less often covered by a CHI plan. Indeed, the coverage rate among non‐eligible young adults was lower than among eligible young adults. Thus, CMUC eligibility has a true impact on the coverage rate for this population.

It is also difficult to draw conclusions about the validity of the threshold when only utilisation data are examined without additional information on health outcomes, offset effects on other types of healthcare (e.g. emergency department visits) or indicators of financial vulnerability. Even though we could not empirically verify whether the average health status differed between the two groups, there is no reason to believe that we would observe a jump or a discontinuity point in the population health status exactly at the CMUC threshold. Recall that we focused our study on the working‐age population and excluded individuals suffering from chronic diseases.

Another limitation of the paper is that the studied population is only representative of the eligible population of the Northern of France and in the urban area of Lille in particular, which undoubtedly has its own particular characteristics. Lille is the fourth‐largest city in France, and inhabitants of the Northern region have the lowest median standard of living in France. The population of the urban area of Lille is quite representative of the French low‐income population (Lecomte and Werquin, 2015). 21The low‐income population of the urban areas of the Northern of France, Lille in particular, is slightly younger than the French low‐income population. Single parent families are over‐represented and the share of the income from social benefits among the poor population of Lille is a bit higher than in the French poor population (Lecomte and Werquin, 2015).


Regarding income manipulation, we checked and rejected the possibility of individuals to manipulate their income at the intensive margin, that is individuals would work less to remain or become eligible for the CMUC plan. In consequence, if one would observe manipulation, it would be at the extensive margin; individuals would work only for a salary below the income threshold. Tables 2 and 6 report similar employment rates in both groups below and above the income threshold. Whilst these figures give us some indication that the extent of this kind of income manipulation is minor and transitory, we cannot fully exclude the existence of manipulation at the extensive margin.

These results suggest that healthcare access programmes for people who are not eligible for the CMUC plan should be focused on populations with a low probability of being covered by a CHI plan, young adults in particular. In France, the ACS programme, which provides a voucher to help ineligible poor households (who are not eligible for CMUC but are eligible for ACS) buy a CHI plan, is quite inefficient; the take‐up rate of this programme is very low, particularly among the uncovered populations. It would certainly be more efficient to target these populations through subsidised health insurance plans specifically designed for their needs by the public health insurance authorities. Indeed, considering the complexity of the offered health insurance plans and the distortion introduced by the ACS voucher, there is no guarantee that the CHI market spontaneously provides such plans. The French government seems to follow this strategy; in 2015, it invited private insurance companies to tender complementary health plans with specific conditions in order to increase ACS take‐up. We could evaluate in future work the impact of this programme adjustment on health insurance coverage rates and healthcare access in the populations with an income above the CMUC threshold, particularly among young adults.

## Supporting information

Figure S1.
