# Supplementary material for: The Impact of the Eligibility Threshold of a French Means‐Tested Health Insurance Programme on Doctor Visits: A Regression Discontinuity Analysis
Source: Health Econ. 2017 Mar 20;26(12):e17–34. doi: 10.1002/hec.3464 (PMC5811792; doi:10.1002/hec.3464)
Supplement: Supplementary file 1 — Figure S1. [file HEC-26-e17-s001.docx]

1. Appendixes

##### 1.1 Measurement errors and regression discontinuity analysis

The definition of eligibility for the CMUC plan is not straightforward. Individuals can apply for the CMUC at any time of the year, and the income from the 12 months prior to application is used to assess eligibility. On the basis of the definition of a CMUC recipient (an individual is considered to benefit from CMUC for an entire year t if she or he is covered by the free plan for at least 6 months), a person is considered to be eligible in year t if she or he is eligible at least once over the period of one year covering 6 months before and 6 months after January 1st of that year t (=2008, 2009). We assume here that a person is necessarily eligible between two spells of CMUC coverage during one year.

In our data, we only observed $Z_{0}$ (for notation simplification, we omit the individual and the year subscript), the income used for the assessment of eligibility on the 1st of January, that is in month m=0. An exact measure of eligibility would suppose that we could observe eligibility for each month from (m-6) to (m+5). An individual is indeed eligible in year t if she or he is eligible at least 1 month over the period of 12 months around the 1st of January, that is, if $Z^{*}=\min\left\{ Z_{m} \right\}_{m\in\left\{ -6,\ldots,5 \right\}}$, is below the eligibility threshold. The following relationship is found between the observed eligibility income $Z_{0}$ and the true eligibility income, $Z^{*}$:

$Z^{*}=aZ_{0}+\left( 1-a \right)\left( Z_{0}-z \right)$ (A.1)

where z> 0 and *a* is a binary indicator (a=0,1). One can rewrite identity A.1 as follows:

$Z_{0}=aZ^{*}+(1-a)Z$ (A.2)

where $Z=\left( Z^{*}+z \right)$represents the erroneous eligibility income. Equation A.2 means that we observe income used for eligibility with error for part of the population and without error for the other part. Put differently, the equation implies that the measurement error in Z is not a systematic error. Indeed, Battistin *et al*. (2009) show that under this measurement error specification, if we can assume that the measurement error is orthogonal to the treatment and the outcome variables and under the assumption that we observe the true eligible income for part of the population, the identification strategy presented in section 4 remains valid.

1.2 No manipulation of the running variable

We checked for the absence of manipulation of the selection variable around the eligibility threshold. To test this hypothesis, we present the distribution of income per CU (normalised by subtracting the eligibility thresholds for 2007 and 2008) around the eligibility threshold. Figure A1 shows a histogram of the distribution of income per CU. A negative value indicates that the income is below the threshold and that the individual is eligible for CMUC. This graph shows the number of observations in each class of 200 Euros wide. An accumulation of observations just below the threshold may indicate the presence of manipulation. In view of the income distribution, it does not seem that individuals manipulate their earnings to come in below the threshold, as there are peaks both to the right and left of the threshold.

McCrary (2008) proposed a simple two-step procedure to check if there is a discontinuity in the density of the income variable. In the first step, the income variable is partitioned in a class of width b and the frequency of observations in each class is calculated. Then, the histogram is smoothed using local linear regressions. The value of income in the middle of each class is treated as an explanatory variable, and the number of observations in each class is treated as the dependent variable. To test the potential discontinuity in the income density, the local linear regression is conducted separately on the right and left of the discontinuity point (CMUC eligibility cut-off point), and a triangular kernel is used with a window of width h, defining which observations are included in the regression. The parameter of interest is the difference in the logarithm of the density function f(z), just below and just above the threshold c: $\hat{\theta}=\ln\lim_{z\to c^{-}} f\left( z \right)-\ln\lim_{z\to c^{+}} f\left( z \right)=ln\hat{f}^{-}-ln\hat{f}^{+}$. A positive and significant difference θ indicates the presence of manipulation around the income threshold. McCrary (2008) showed that the estimator $\theta=lnf^{-}-lnf^{+}$ is asymptotically normal.

Figure A1 Distribution of income per CU around the cut-off point c

(pooled data for the years 2008 and 2009, bin size=200 Euros).


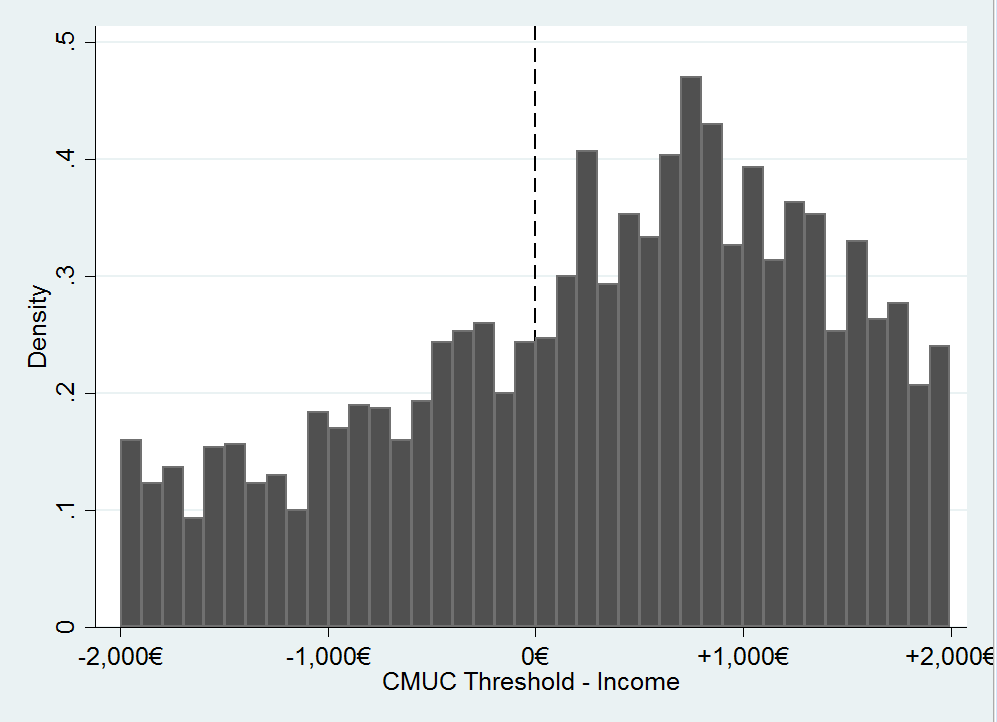


Note: As the eligibility threshold differs in 2007 and 2008, income per CU is normalised by subtracting the corresponding eligibility cut-off point. A negative value indicates that income is below the threshold and the individual is eligible for CMUC. Our sample consists of individuals with a normalised income per CU between ± 2,000 Euros around the eligibility threshold.

Figure A2 shows the estimated graph and Table A1 the estimated results of the density test. Estimates indicate that the difference in the logarithm of the density function to the right and left of the threshold is equal to 0.1749 with a standard deviation equal to 0.1339. The test rejects the presence of a discontinuity in the density function at the cut-off point (t-statistic = 1.30). According to McCrary (2008), the choice of class size b in the first stage of the estimation does not matter, but it is necessary to choose the estimation window h in the second stage to ensure a good test performance. Table A1 shows test results for multiple windows h and a fixed size class b equal to 37 Euros. In all cases, the hypothesis of no discontinuity in the density at the threshold c was not rejected.

Table A1 Estimates of the manipulation test of the selection variable (McCrary 2008)

| Bandwidth (h)  (in thousand Euros) | Automatic  (0.72) | 2.00 | 1.00 |
| --- | --- | --- | --- |
| $\hat{\theta}$ | 0.1749 | -0.1636 | 0.137 |
| Standard deviation | 0.1339 | 0.1154 | 0.1116 |
| t-statistic | 1.30 | -1.42 | 1.22 |

*Notes:* $\hat{\theta}=ln\hat{f}^{-}ln\hat{f}^{+}$estimates the discontinuity in the density function around the eligibility threshold. A positive and significant statistic indicates the presence of significant manipulation of income. The smoothing parameter (h) is automatically the one proposed by McCrary (2008). The size of each class (b) is equal to 37 Euros. Estimates were performed using Stata command "DCdensity.ado", developed by McCrary (2008).

Figure A2 Estimated density function (McCrary test)

*Notes:* Points are estimated densities with a class size of 37 Euros. The lines are estimates of local linear regressions using a triangular kernel function and a smoothing parameter of 51 Euros. Standard errors are calculated as in McCrary (2008)

1.3 Sensitivity analysis of the income specification

Tables A2 –12 report the estimates of equations:

| ${E[V}_{it}\left\vert X_{it},{eliCMUC}_{it},Z_{it-1} \right]=a+b*X_{it}+d*{eliCMUC}_{it}+{\left( 1-{eliCMUC}_{it} \right)*h}_{g2}\left( Z_{it-1} \right)+{{eliCMUC}_{it}*h}_{g1}\left( Z_{it-1} \right)+E[w_{it}\left\vert X_{it},{eliCMUC}_{it},Z_{it-1} \right]$ | (2) |
| --- | --- |
| ${E[N}_{it}\vert X_{it},{eliCMUC}_{it},Z_{it-1}]=exp[A+B*X_{it}+D*{eliCMUC}_{it}+ {{eliCMUC}_{it}*H}_{g1}\left( Z_{it-1} \right)+{(1-{eliCMUC}_{it})*H}_{g2}\left( Z_{it-1} \right)+{E(W}_{it}\left\vert X_{it},{eliCMUC}_{it},Z_{it-1}) \right]$ | (2’) |

Where $N_{it}$ is the number of visits of $i$ in year $t$ and ${eliCMUC}_{it}=I\left( Z_{it-1}<c_{it} \right)$ in equation (2) and $V_{it}(=0,1)$ is a binary variable indicating if the individual $i$ visited a doctor at least once during year $t$ in equation (2’). D and d are our parameters of interest. We add polynomial functions of household income, $H_{g1}$($Z_{it-1}$), $h_{g1}$($Z_{it-1}$) and $H_{g2}$($Z_{it-1}$), $h_{g2}$($Z_{it-1}$) and $X_{it}$ a matrix of control variables. $w_{it}$, $W_{it}$ are the unobserved error components with ${E(W}_{it}|X_{it},{eliCMUC}_{it},Z_{it-1})=0$ and ${E(w}_{it}|X_{it},{eliCMUC}_{it},Z_{it-1})=0$.

Estimations are run on the full sample and on the under 30 populations. We report estimates for the total number of doctor visits and for the number of GP visits and specialist visits separately.

*1.3.1 Number of doctor visits (total)*

Table A2. Impact of CMUC eligibility on total number of visits (Full sample)

|  | Without controls (a) | | | With controls (b) | | |
| --- | --- | --- | --- | --- | --- | --- |
|  | (1) | (2) | (3) | (1) | (2) | (3) |
|  |  |  |  |  |  |  |
| eliCMUCI *(irr)* | 1.003 | 0.964 | 0.969 | 1.010 | 0.978 | 0.985 |
| *se* | (0.1103) | (0.1098) | (0.1108) | (0.1101) | (0.1102) | (0.1114) |
| *p-value* | 0.9773 | 0.7453 | 0.7841 | 0.9277 | 0.8436 | 0.8922 |
| Eli*Income *(irr)* |  | 0.885 |  |  | 0.914 |  |
| *se* |  | (0.0680) |  |  | (0.0690) |  |
| *p-value* |  | 0.1117 |  |  | 0.2356 |  |
| No-eli*Income *(irr)* |  | 0.985 |  |  | 0.996 |  |
| *se* |  | (0.0564) |  |  | (0.0561) |  |
| *p-value* |  | 0.7959 |  |  | 0.9393 |  |
| Income *(irr)*I | 0.950 |  | 0.936 | 0.967 |  | 0.957 |
| *se* | (0.0434) |  | (0.0445) | (0.0435) |  | (0.0449) |
| *p-value* | 0.2583 |  | 0.1664 | 0.4621 |  | 0.3516 |
| Income² *(irr)* |  |  | 1.020 |  |  | 1.014 |
| *se* |  |  | (0.0208) |  |  | (0.0202) |
| *p-value* |  |  | 0.3436 |  |  | 0.4800 |
|  |  |  |  |  |  |  |
| N | 2,232 | 2,232 | 2,232 | 2,232 | 2,232 | 2,232 |

*Notes:* All regressions are negative binomial models; the exponential of the estimated coefficient is reported, incident rate ratio, IRR. (a) Regressions include family income and a year dummy for 2008. (b) Regressions include family income, a year dummy for 2008, individual and family characteristics. Robust standard errors (s.e.) are in parentheses. Statistical significance levels: * p<=10%; ** p<=5%; *** p<=1%.

Table A3. Impact of CMUC eligibility on total number of visits (Under 30)

|  | Without controls (a) | | | With controls (b) | | |
| --- | --- | --- | --- | --- | --- | --- |
|  | (1) | (2) | (3) | (1) | (2) | (3) |
|  |  |  |  |  |  |  |
| eliCMUCI *(irr)* | 1.599** | 1.541** | 1.542** | 1.584** | 1.525** | 1.525** |
| *se* | (0.3438) | (0.3298) | (0.3299) | (0.3422) | (0.3241) | (0.3242) |
| *p-value* | 0.0291 | 0.0433 | 0.0432 | 0.0334 | 0.0473 | 0.0469 |
| Eli*Income *(irr)* |  | 1.075 |  |  | 1.096 |  |
| *se* |  | (0.1555) |  |  | (0.1477) |  |
| *p-value* |  | 0.6192 |  |  | 0.4963 |  |
| Noeli*Income *(irr)* |  | 1.248* |  |  | 1.282** |  |
| *se* |  | (0.1516) |  |  | (0.1579) |  |
| *p-value* |  | 0.0685 |  |  | 0.0438 |  |
| Income *(irr)*I | 1.182* |  | 1.159 | 1.211** |  | 1.187* |
| *se* | (0.1100) |  | (0.1087) | (0.1117) |  | (0.1083) |
| *p-value* | 0.0726 |  | 0.1148 | 0.0381 |  | 0.0603 |
| Income² *(irr)* |  |  | 1.029 |  |  | 1.030 |
| *se* |  |  | (0.0418) |  |  | (0.0399) |
| *p-value* |  |  | 0.4824 |  |  | 0.4429 |
|  |  |  |  |  |  |  |
| N | 532 | 532 | 532 | 532 | 532 | 532 |

*Notes:* All regressions are negative binomial models; the exponential of the estimated coefficient is reported, incident rate ratio, IRR. (a) Regressions include family income and a year dummy for 2008. (b) Regressions include family income, a year dummy for 2008, individual and family characteristics. Robust standard errors (s.e.) are in parentheses. Statistical significance levels: * p<=10%; ** p<=5%; *** p<=1%.

*1.3.2 Conditional number of doctor visits (total)*

Table A4. Impact of CMUC eligibility on total conditional number of visits (Full sample)

|  | Without controls (a) | | | | | | With controls (b) | | | | | |  |
| --- | --- | --- | --- | --- | --- | --- | --- | --- | --- | --- | --- | --- | --- |
|  | (1) | | (2) | | (3) | | (1) | | (2) | | (3) | |  |
|  |  | |  | |  | |  | |  | |  | |  |
| eliCMUCI *(irr)* | | 1.011 | | 0.940 | | 0.944 | | 1.022 | | 0.958 | | 0.962 | |
| *se* | | (0.0976) | | (0.0955) | | (0.0960) | | (0.0976) | | (0.0965) | | (0.0971) | |
| *p-value* | | 0.9103 | | 0.5421 | | 0.5676 | | 0.8192 | | 0.6703 | | 0.7000 | |
| Eli*Income *(irr)* | |  | | 0.840** | |  | |  | | 0.871** | |  | |
| *se* | |  | | (0.0574) | |  | |  | | (0.0590) | |  | |
| *p-value* | |  | | 0.0106 | |  | |  | | 0.0410 | |  | |
| Noeli*Income *(irr)* | |  | | 1.030 | |  | |  | | 1.042 | |  | |
| *se* | |  | | (0.0510) | |  | |  | | (0.0505) | |  | |
| *p-value* | |  | | 0.5472 | |  | |  | | 0.3940 | |  | |
| Income *(irr)*I | | 0.959 | |  | | 0.932* | | 0.980 | |  | | 0.955 | |
| *se* | | (0.0386) | |  | | (0.0391) | | (0.0388) | |  | | (0.0398) | |
| *p-value* | | 0.2998 | |  | | 0.0953 | | 0.6016 | |  | | 0.2676 | |
| Income² *(irr)* | |  | |  | | 1.041** | |  | |  | | 1.036** | |
| *se* | |  | |  | | (0.0185) | |  | |  | | (0.0180) | |
| *p-value* | |  | |  | | 0.0222 | |  | |  | | 0.0418 | |
|  | |  | |  | |  | |  | |  | |  | |
| N | | 1,847 | | 1,847 | | 1,847 | | 1,847 | | 1,847 | | 1,847 | |

*Notes:* All regressions are negative binomial models; the exponential of the estimated coefficient is reported, incident rate ratio, IRR. (a) Regressions include family income and a year dummy for 2008. (b) Regressions include family income, a year dummy for 2008, individual and family characteristics. Robust standard errors (s.e.) are in parentheses. Statistical significance levels: * p<=10%; ** p<=5%; *** p<=1%.

Table A5. Impact of CMUC eligibility on total conditional number of visits (Under 30)

|  | Without controls (a) | | | | | | With controls (b) | | | | | |  |
| --- | --- | --- | --- | --- | --- | --- | --- | --- | --- | --- | --- | --- | --- |
|  | (1) | | (2) | | (3) | | (1) | | (2) | | (3) | |  |
|  |  | |  | |  | |  | |  | |  | |  |
| eliCMUCI *(irr)* | | 1.514** | | 1.455** | | 1.450** | | 1.503** | | 1.443** | | 1.438** | |
| *se* | | (0.2834) | | (0.2758) | | (0.2751) | | (0.2761) | | (0.2663) | | (0.2656) | |
| *p-value* | | 0.0265 | | 0.0479 | | 0.0499 | | 0.0267 | | 0.0470 | | 0.0491 | |
| Eli*Income *(irr)* | |  | | 1.058 | |  | |  | | 1.071 | |  | |
| *se* | |  | | (0.1406) | |  | |  | | (0.1334) | |  | |
| *p-value* | |  | | 0.6699 | |  | |  | | 0.5806 | |  | |
| Noeli*Income *(irr)* | |  | | 1.279** | |  | |  | | 1.301** | |  | |
| *se* | |  | | (0.1337) | |  | |  | | (0.1351) | |  | |
| *p-value* | |  | | 0.0184 | |  | |  | | 0.0112 | |  | |
| Income *(irr)*I | | 1.191** | |  | | 1.163* | | 1.209** | |  | | 1.180** | |
| *se* | | (0.0983) | |  | | (0.0983) | | (0.0972) | |  | | (0.0961) | |
| *p-value* | | 0.0340 | |  | | 0.0734 | | 0.0183 | |  | | 0.0418 | |
| Income² *(irr)* | |  | |  | | 1.039 | |  | |  | | 1.040 | |
| *se* | |  | |  | | (0.0372) | |  | |  | | (0.0355) | |
| *p-value* | |  | |  | | 0.2802 | |  | |  | | 0.2466 | |
|  | |  | |  | |  | |  | |  | |  | |
| N | | 429 | | 429 | | 429 | | 429 | | 429 | | 429 | |

*Notes:* All regressions are negative binomial models; the exponential of the estimated coefficient is reported, incident rate ratio, IRR. (a) Regressions include family income and a year dummy for 2008. (b) Regressions include family income, a year dummy for 2008, individual and family characteristics. Robust standard errors (s.e.) are in parentheses. Statistical significance levels: * p<=10%; ** p<=5%; *** p<=1%.

*1.3.3 Probability of seeing at least once a doctor (total)*

Table A6. Impact of CMUC eligibility on the probability of seeing a doctor (Full sample)

|  | Without controls (a) | | | With controls (b) | | |
| --- | --- | --- | --- | --- | --- | --- |
|  | (1) | (2) | (3) | (1) | (2) | (3) |
|  |  |  |  |  |  |  |
| eliCMUCI *(od)* | 0.945 | 1.207 | 1.217 | 0.977 | 1.238 | 1.252 |
| *se* | (0.2878) | (0.4001) | (0.4000) | (0.3051) | (0.4192) | (0.4216) |
| *p-value* | 0.8521 | 0.5704 | 0.5501 | 0.9397 | 0.5290 | 0.5051 |
| Eli*Income *(od)* |  | 1.380 |  |  | 1.368 |  |
| *se* |  | (0.2818) |  |  | (0.2879) |  |
| *p-value* |  | 0.1151 |  |  | 0.1362 |  |
| Noeli*Income *(od)* |  | 0.782 |  |  | 0.797 |  |
| *se* |  | (0.1213) |  |  | (0.1262) |  |
| *p-value* |  | 0.1121 |  |  | 0.1523 |  |
| Income *(od)* | 0.936 |  | 1.040 | 0.943 |  | 1.047 |
| *se* | (0.1173) |  | (0.1312) | (0.1210) |  | (0.1366) |
| *p-value* | 0.5980 |  | 0.7587 | 0.6482 |  | 0.7240 |
| Income² *(od)* |  |  | 0.885** |  |  | 0.890** |
| *se* |  |  | (0.0466) |  |  | (0.0480) |
| *p-value* |  |  | 0.0205 |  |  | 0.0303 |
|  |  |  |  |  |  |  |
| N | 2,232 | 2,232 | 2,232 | 2,232 | 2,232 | 2,232 |

*Notes:* All regressions are logit models; Odds ratio are reported (OR). (a) Regressions include family income and a year dummy for 2008. (b) Regressions include family income, a year dummy for 2008, individual and family characteristics. Robust standard errors (s.e.) are in parentheses. Statistical significance levels: * p<=10%; ** p<=5%; *** p<=1%.

Table A7. Impact of CMUC eligibility on the probability of seeing a doctor (Under 30)

|  | Without controls (a) | | | With controls (b) | | |
| --- | --- | --- | --- | --- | --- | --- |
|  | (1) | (2) | (3) | (1) | (2) | (3) |
|  |  |  |  |  |  |  |
| eliCMUCI *(od)* | 1.265 | 1.370 | 1.392 | 1.380 | 1.439 | 1.475 |
| *se* | (0.7910) | (0.8828) | (0.8937) | (0.9077) | (0.9541) | (0.9756) |
| *p-value* | 0.7066 | 0.6250 | 0.6060 | 0.6244 | 0.5829 | 0.5569 |
| Eli*Income *(od)* |  | 1.064 |  |  | 1.047 |  |
| *se* |  | (0.4503) |  |  | (0.4475) |  |
| *p-value* |  | 0.8832 |  |  | 0.9149 |  |
| Noeli*Income *(od)* |  | 0.878 |  |  | 0.942 |  |
| *se* |  | (0.2770) |  |  | (0.3182) |  |
| *p-value* |  | 0.6795 |  |  | 0.8606 |  |
| Income *(od)* | 0.928 |  | 0.973 | 0.972 |  | 1.005 |
| *se* | (0.2387) |  | (0.2532) | (0.2628) |  | (0.2702) |
| *p-value* | 0.7717 |  | 0.9169 | 0.9170 |  | 0.9864 |
| Income² *(od)* |  |  | 0.953 |  |  | 0.967 |
| *se* |  |  | (0.1066) |  |  | (0.1123) |
| *p-value* |  |  | 0.6696 |  |  | 0.7695 |
|  |  |  |  |  |  |  |
| N | 532 | 532 | 532 | 532 | 532 | 532 |

*Notes:* All regressions are logit models; Odds ratio are reported (OR). (a) Regressions include family income and a year dummy for 2008. (b) Regressions include family income, a year dummy for 2008, individual and family characteristics. Robust standard errors (s.e.) are in parentheses. Statistical significance levels: * p<=10%; ** p<=5%; *** p<=1%.

*1.3.4 Number of GP visits*

Table A8. Impact of CMUC eligibility on number of GP visits (Full sample)

|  | Without controls (a) | | | | | | With controls (b) | | | | | |  |
| --- | --- | --- | --- | --- | --- | --- | --- | --- | --- | --- | --- | --- | --- |
|  | (1) | | (2) | | (3) | | (1) | | (2) | | (3) | |  |
|  |  | |  | |  | |  | |  | |  | |  |
| eliCMUCI *(irr)* | | 0.954 | | 0.933 | | 0.936 | | 0.972 | | 0.958 | | 0.962 | |
| *se* | | (0.1054) | | (0.1072) | | (0.1079) | | (0.1068) | | (0.1093) | | (0.1103) | |
| *p-value* | | 0.6678 | | 0.5438 | | 0.5638 | | 0.7989 | | 0.7073 | | 0.7374 | |
| Eli*Income *(irr)* | |  | | 0.902 | |  | |  | | 0.934 | |  | |
| *se* | |  | | (0.0713) | |  | |  | | (0.0724) | |  | |
| *p-value* | |  | | 0.1924 | |  | |  | | 0.3819 | |  | |
| Noeli*Income *(irr)* | |  | | 0.959 | |  | |  | | 0.973 | |  | |
| *se* | |  | | (0.0550) | |  | |  | | (0.0550) | |  | |
| *p-value* | |  | | 0.4669 | |  | |  | | 0.6273 | |  | |
| Income *(irr)*I | | 0.939 | |  | | 0.932 | | 0.960 | |  | | 0.955 | |
| *se* | | (0.0431) | |  | | (0.0450) | | (0.0433) | |  | | (0.0453) | |
| *p-value* | | 0.1703 | |  | | 0.1426 | | 0.3606 | |  | | 0.3366 | |
| Income² *(irr)* | |  | |  | | 1.011 | |  | |  | | 1.006 | |
| *se* | |  | |  | | (0.0210) | |  | |  | | (0.0204) | |
| *p-value* | |  | |  | | 0.5970 | |  | |  | | 0.7702 | |
|  | |  | |  | |  | |  | |  | |  | |
| N | | 2,232 | | 2,232 | | 2,232 | | 2,232 | | 2,232 | | 2,232 | |

*Notes:* All regressions are negative binomial models; the exponential of the estimated coefficient is reported, incident rate ratio, IRR. (a) Regressions include family income and a year dummy for 2008. (b) Regressions include family income, a year dummy for 2008, individual and family characteristics. Robust standard errors (s.e.) are in parentheses. Statistical significance levels: * p<=10%; ** p<=5%; *** p<=1%.

Table A9. Impact of CMUC eligibility on number of GP visits (Under 30)

|  | Without controls (a) | | | | | | With controls (b) | | | | | |  |
| --- | --- | --- | --- | --- | --- | --- | --- | --- | --- | --- | --- | --- | --- |
|  | (1) | | (2) | | (3) | | (1) | | (2) | | (3) | |  |
|  |  | |  | |  | |  | |  | |  | |  |
| eliCMUCI *(irr)* | | 1.344 | | 1.334 | | 1.340 | | 1.323 | | 1.308 | | 1.314 | |
| *se* | | (0.2998) | | (0.2954) | | (0.2973) | | (0.2974) | | (0.2872) | | (0.2890) | |
| *p-value* | | 0.1848 | | 0.1932 | | 0.1864 | | 0.2124 | | 0.2217 | | 0.2140 | |
| Eli*Income *(irr)* | |  | | 1.096 | |  | |  | | 1.107 | |  | |
| *se* | |  | | (0.1560) | |  | |  | | (0.1452) | |  | |
| *p-value* | |  | | 0.5176 | |  | |  | | 0.4395 | |  | |
| Noeli*Income *(irr)* | |  | | 1.132 | |  | |  | | 1.164 | |  | |
| *se* | |  | | (0.1388) | |  | |  | | (0.1465) | |  | |
| *p-value* | |  | | 0.3103 | |  | |  | | 0.2279 | |  | |
| Income *(irr)*I | | 1.119 | |  | | 1.117 | | 1.143 | |  | | 1.138 | |
| *se* | | (0.1042) | |  | | (0.1049) | | (0.1060) | |  | | (0.1039) | |
| *p-value* | | 0.2266 | |  | | 0.2367 | | 0.1510 | |  | | 0.1557 | |
| Income² *(irr)* | |  | |  | | 1.002 | |  | |  | | 1.006 | |
| *se* | |  | |  | | (0.0398) | |  | |  | | (0.0381) | |
| *p-value* | |  | |  | | 0.9547 | |  | |  | | 0.8806 | |
|  | |  | |  | |  | |  | |  | |  | |
| N | | 532 | | 532 | | 532 | | 532 | | 532 | | 532 | |

*Notes:* All regressions are negative binomial models; the exponential of the estimated coefficient is reported, incident rate ratio, IRR. (a) Regressions include family income and a year dummy for 2008. (b) Regressions include family income, a year dummy for 2008, individual and family characteristics. Robust standard errors (s.e.) are in parentheses. Statistical significance levels: * p<=10%; ** p<=5%; *** p<=1%.

*1.3.5 Conditional number of GP visits*

Table A10. Impact of CMUC eligibility on conditional number of GP visits (Full sample)

|  | Without controls (a) | | | With controls (b) | | |
| --- | --- | --- | --- | --- | --- | --- |
|  | (1) | (2) | (3) | (1) | (2) | (3) |
|  |  |  |  |  |  |  |
| eliCMUCI *(irr)* | 0.932 | 0.895 | 0.894 | 0.952 | 0.923 | 0.922 |
| *se* | (0.0868) | (0.0886) | (0.0888) | (0.0882) | (0.0912) | (0.0915) |
| *p-value* | 0.4489 | 0.2607 | 0.2589 | 0.5985 | 0.4161 | 0.4132 |
| Eli*Income *(irr)* |  | 0.867** |  |  | 0.905 |  |
| *se* |  | (0.0602) |  |  | (0.0624) |  |
| *p-value* |  | 0.0395 |  |  | 0.1477 |  |
| Noeli*Income *(irr)* |  | 0.975 |  |  | 0.990 |  |
| *se* |  | (0.0463) |  |  | (0.0462) |  |
| *p-value* |  | 0.5984 |  |  | 0.8323 |  |
| Income *(irr)*I | 0.935* |  | 0.920** | 0.960 |  | 0.947 |
| *se* | (0.0366) |  | (0.0384) | (0.0370) |  | (0.0391) |
| *p-value* | 0.0881 |  | 0.0446 | 0.2839 |  | 0.1847 |
| Income² *(irr)* |  |  | 1.025 |  |  | 1.019 |
| *se* |  |  | (0.0182) |  |  | (0.0178) |
| *p-value* |  |  | 0.1577 |  |  | 0.2699 |
|  |  |  |  |  |  |  |
| N | 1,770 | 1,770 | 1,770 | 1,770 | 1,770 | 1,770 |

*Notes:* All regressions are negative binomial models; the exponential of the estimated coefficient is reported, incident rate ratio, IRR. (a) Regressions include family income and a year dummy for 2008. (b) Regressions include family income, a year dummy for 2008, individual and family characteristics. Robust standard errors (s.e.) are in parentheses. Statistical significance levels: * p<=10%; ** p<=5%; *** p<=1%.

Table A11. Impact of CMUC eligibility on conditional number of GP visits (Under 30)

|  | Without controls (a) | | | With controls (b) | | |
| --- | --- | --- | --- | --- | --- | --- |
|  | (1) | (2) | (3) | (1) | (2) | (3) |
|  |  |  |  |  |  |  |
| eliCMUCI *(irr)* | 1.268 | 1.237 | 1.233 | 1.245 | 1.211 | 1.207 |
| *se* | (0.2359) | (0.2337) | (0.2339) | (0.2295) | (0.2247) | (0.2248) |
| *p-value* | 0.2015 | 0.2609 | 0.2689 | 0.2337 | 0.3016 | 0.3114 |
| Eli*Income *(irr)* |  | 1.038 |  |  | 1.039 |  |
| *se* |  | (0.1325) |  |  | (0.1247) |  |
| *p-value* |  | 0.7690 |  |  | 0.7506 |  |
| Noeli*Income *(irr)* |  | 1.164 |  |  | 1.179* |  |
| *se* |  | (0.1157) |  |  | (0.1176) |  |
| *p-value* |  | 0.1254 |  |  | 0.0979 |  |
| Income *(irr)*I | 1.116 |  | 1.099 | 1.125 |  | 1.106 |
| *se* | (0.0874) |  | (0.0891) | (0.0865) |  | (0.0867) |
| *p-value* | 0.1614 |  | 0.2451 | 0.1249 |  | 0.1978 |
| Income² *(irr)* |  |  | 1.025 |  |  | 1.027 |
| *se* |  |  | (0.0342) |  |  | (0.0328) |
| *p-value* |  |  | 0.4666 |  |  | 0.3992 |
|  |  |  |  |  |  |  |
| N | 409 | 409 | 409 | 409 | 409 | 409 |

*Notes:* All regressions are negative binomial models; the exponential of the estimated coefficient is reported, incident rate ratio, IRR. (a) Regressions include family income and a year dummy for 2008. (b) Regressions include family income, a year dummy for 2008, individual and family characteristics. Robust standard errors (s.e.) are in parentheses. Statistical significance levels: * p<=10%; ** p<=5%; *** p<=1%.

*1.3.6 Probability of seeing at least once a GP*

Table A12. Impact of CMUC eligibility on the probability of seeing a GP (Full sample)

|  | Without controls (a) | | | | | | With controls (b) | | | | | |  |
| --- | --- | --- | --- | --- | --- | --- | --- | --- | --- | --- | --- | --- | --- |
|  | (1) | | (2) | | (3) | | (1) | | (2) | | (3) | |  |
|  |  | |  | |  | |  | |  | |  | |  |
| eliCMUCI *(od)* | | 1.113 | | 1.256 | | 1.283 | | 1.144 | | 1.270 | | 1.301 | |
| *se* | | (0.3245) | | (0.3838) | | (0.3923) | | (0.3399) | | (0.3942) | | (0.4051) | |
| *p-value* | | 0.7146 | | 0.4557 | | 0.4151 | | 0.6496 | | 0.4414 | | 0.3980 | |
| Eli*Income *(od)* | |  | | 1.230 | |  | |  | | 1.193 | |  | |
| *se* | |  | | (0.2355) | |  | |  | | (0.2344) | |  | |
| *p-value* | |  | | 0.2804 | |  | |  | | 0.3703 | |  | |
| Noeli*Income *(od)* | |  | | 0.923 | |  | |  | | 0.937 | |  | |
| *se* | |  | | (0.1370) | |  | |  | | (0.1406) | |  | |
| *p-value* | |  | | 0.5890 | |  | |  | | 0.6628 | |  | |
| Income *(od)* | | 1.012 | |  | | 1.073 | | 1.011 | |  | | 1.066 | |
| *se* | | (0.1200) | |  | | (0.1288) | | (0.1220) | |  | | (0.1312) | |
| *p-value* | | 0.9182 | |  | | 0.5557 | | 0.9290 | |  | | 0.6009 | |
| Income² *(od)* | |  | |  | | 0.932 | |  | |  | | 0.940 | |
| *se* | |  | |  | | (0.0468) | |  | |  | | (0.0482) | |
| *p-value* | |  | |  | | 0.1609 | |  | |  | | 0.2266 | |
|  | |  | |  | |  | |  | |  | |  | |
| N | | 2,232 | | 2,232 | | 2,232 | | 2,232 | | 2,232 | | 2,232 | |

*Notes:* All regressions are logit models; Odds ratio are reported (OR). (a) Regressions include family income and a year dummy for 2008. (b) Regressions include family income, a year dummy for 2008, individual and family characteristics. Robust standard errors (s.e.) are in parentheses. Statistical significance levels: * p<=10%; ** p<=5%; *** p<=1%.

Table A13. Impact of CMUC eligibility on the probability of seeing a GP (Under 30)

|  | Without controls (a) | | | | | | With controls (b) | | | | | |  |
| --- | --- | --- | --- | --- | --- | --- | --- | --- | --- | --- | --- | --- | --- |
|  | (1) | | (2) | | (3) | | (1) | | (2) | | (3) | |  |
|  |  | |  | |  | |  | |  | |  | |  |
| eliCMUCI *(od)* | | 1.214 | | 1.398 | | 1.451 | | 1.310 | | 1.452 | | 1.517 | |
| *se* | | (0.7171) | | (0.8611) | | (0.8919) | | (0.8026) | | (0.9051) | | (0.9455) | |
| *p-value* | | 0.7421 | | 0.5862 | | 0.5449 | | 0.6599 | | 0.5497 | | 0.5041 | |
| Eli*Income *(od)* | |  | | 1.275 | |  | |  | | 1.248 | |  | |
| *se* | |  | | (0.5100) | |  | |  | | (0.4995) | |  | |
| *p-value* | |  | | 0.5439 | |  | |  | | 0.5799 | |  | |
| Noeli*Income *(od)* | |  | | 0.866 | |  | |  | | 0.931 | |  | |
| *se* | |  | | (0.2636) | |  | |  | | (0.3020) | |  | |
| *p-value* | |  | | 0.6360 | |  | |  | | 0.8257 | |  | |
| Income *(od)* | | 0.979 | |  | | 1.067 | | 1.023 | |  | | 1.099 | |
| *se* | | (0.2424) | |  | | (0.2654) | | (0.2645) | |  | | (0.2809) | |
| *p-value* | | 0.9307 | |  | | 0.7933 | | 0.9286 | |  | | 0.7122 | |
| Income² *(od)* | |  | |  | | 0.904 | |  | |  | | 0.918 | |
| *se* | |  | |  | | (0.0944) | |  | |  | | (0.0987) | |
| *p-value* | |  | |  | | 0.3351 | |  | |  | | 0.4275 | |
|  | |  | |  | |  | |  | |  | |  | |
| N | | 532 | | 532 | | 532 | | 532 | | 532 | | 532 | |

*Notes:* All regressions are logit models; Odds ratio are reported (OR). (a) Regressions include family income and a year dummy for 2008. (b) Regressions include family income, a year dummy for 2008, individual and family characteristics. Robust standard errors (s.e.) are in parentheses. Statistical significance levels: * p<=10%; ** p<=5%; *** p<=1%.

*7.3.7 Number of specialist visits*

Table A14. Impact of CMUC eligibility on number of specialist visits (Full sample)

|  | Without controls (a) | | | | | | With controls (b) | | | | | |  |
| --- | --- | --- | --- | --- | --- | --- | --- | --- | --- | --- | --- | --- | --- |
|  | (1) | | (2) | | (3) | | (1) | | (2) | | (3) | |  |
|  |  | |  | |  | |  | |  | |  | |  |
| eliCMUCI *(irr)* | | 1.202 | | 1.067 | | 1.084 | | 1.159 | | 1.047 | | 1.063 | |
| *se* | | (0.2436) | | (0.2291) | | (0.2316) | | (0.2322) | | (0.2229) | | (0.2257) | |
| *p-value* | | 0.3650 | | 0.7642 | | 0.7059 | | 0.4616 | | 0.8282 | | 0.7731 | |
| Eli*Income *(irr)* | |  | | 0.805 | |  | |  | | 0.837 | |  | |
| *se* | |  | | (0.1135) | |  | |  | | (0.1162) | |  | |
| *p-value* | |  | | 0.1233 | |  | |  | | 0.2011 | |  | |
| Noeli*Income *(irr)* | |  | | 1.093 | |  | |  | | 1.089 | |  | |
| *se* | |  | | (0.1127) | |  | |  | | (0.1103) | |  | |
| *p-value* | |  | | 0.3861 | |  | |  | | 0.3991 | |  | |
| Income *(irr)*I | | 0.986 | |  | | 0.945 | | 0.997 | |  | | 0.962 | |
| *se* | | (0.0830) | |  | | (0.0821) | | (0.0828) | |  | | (0.0829) | |
| *p-value* | | 0.8642 | |  | | 0.5151 | | 0.9717 | |  | | 0.6506 | |
| Income² *(irr)* | |  | |  | | 1.057 | |  | |  | | 1.048 | |
| *se* | |  | |  | | (0.0387) | |  | |  | | (0.0376) | |
| *p-value* | |  | |  | | 0.1275 | |  | |  | | 0.1873 | |
|  | |  | |  | |  | |  | |  | |  | |
| N | | 2,232 | | 2,232 | | 2,232 | | 2,232 | | 2,232 | | 2,232 | |

*Notes:* All regressions are negative binomial models; the exponential of the estimated coefficient is reported, incident rate ratio, IRR. (a) Regressions include family income and a year dummy for 2008. (b) Regressions include family income, a year dummy for 2008, individual and family characteristics. Robust standard errors (s.e.) are in parentheses. Statistical significance levels: * p<=10%; ** p<=5%; *** p<=1%.

Table A15. Impact of CMUC eligibility on number of specialist visits (Under 30)

|  | Without controls (a) | | | | | | With controls (b) | | | | | |  |
| --- | --- | --- | --- | --- | --- | --- | --- | --- | --- | --- | --- | --- | --- |
|  | (1) | | (2) | | (3) | | (1) | | (2) | | (3) | |  |
|  |  | |  | |  | |  | |  | |  | |  |
| eliCMUCI *(irr)* | | 3.058*** | | 2.698** | | 2.617** | | 3.247*** | | 2.880*** | | 2.797*** | |
| *se* | | (1.1705) | | (1.0435) | | (1.0075) | | (1.2162) | | (1.0939) | | (1.0560) | |
| *p-value* | | 0.0035 | | 0.0103 | | 0.0125 | | 0.0017 | | 0.0054 | | 0.0064 | |
| Eli*Income *(irr)* | |  | | 1.011 | |  | |  | | 1.060 | |  | |
| *se* | |  | | (0.2662) | |  | |  | | (0.2804) | |  | |
| *p-value* | |  | | 0.9679 | |  | |  | | 0.8260 | |  | |
| Noeli*Income *(irr)* | |  | | 1.787*** | |  | |  | | 1.884*** | |  | |
| *se* | |  | | (0.4014) | |  | |  | | (0.4197) | |  | |
| *p-value* | |  | | 0.0097 | |  | |  | | 0.0045 | |  | |
| Income *(irr)*I | | 1.449** | |  | | 1.327* | | 1.521** | |  | | 1.396** | |
| *se* | | (0.2547) | |  | | (0.2261) | | (0.2656) | |  | | (0.2359) | |
| *p-value* | | 0.0349 | |  | | 0.0970 | | 0.0164 | |  | | 0.0481 | |
| Income² *(irr)* | |  | |  | | 1.137* | |  | |  | | 1.136* | |
| *se* | |  | |  | | (0.0844) | |  | |  | | (0.0851) | |
| *p-value* | |  | |  | | 0.0842 | |  | |  | | 0.0881 | |
|  | |  | |  | |  | |  | |  | |  | |
| N | | 532 | | 532 | | 532 | | 532 | | 532 | | 532 | |

*Notes:* All regressions are negative binomial models; the exponential of the estimated coefficient is reported, incident rate ratio, IRR. (a) Regressions include family income and a year dummy for 2008. (b) Regressions include family income, a year dummy for 2008, individual and family characteristics. Robust standard errors (s.e.) are in parentheses. Statistical significance levels: * p<=10%; ** p<=5%; *** p<=1%.

*1.3.8 Conditional number of specialist visits*

Table A9. Impact of CMUC eligibility on conditional number of specialist visits

(Full sample)

|  | Without controls (a) | | | | | | With controls (b) | | | | | |  |
| --- | --- | --- | --- | --- | --- | --- | --- | --- | --- | --- | --- | --- | --- |
|  | (1) | | (2) | | (3) | | (1) | | (2) | | (3) | |  |
|  |  | |  | |  | |  | |  | |  | |  |
| eliCMUCI *(irr)* | | 1.074 | | 1.007 | | 1.009 | | 1.084 | | 1.023 | | 1.024 | |
| *se* | | (0.1492) | | (0.1503) | | (0.1490) | | (0.1510) | | (0.1549) | | (0.1538) | |
| *p-value* | | 0.6054 | | 0.9611 | | 0.9523 | | 0.5634 | | 0.8831 | | 0.8756 | |
| Eli*Income *(irr)* | |  | | 0.873 | |  | |  | | 0.900 | |  | |
| *se* | |  | | (0.0882) | |  | |  | | (0.0940) | |  | |
| *p-value* | |  | | 0.1782 | |  | |  | | 0.3147 | |  | |
| Noeli*Income *(irr)* | |  | | 1.050 | |  | |  | | 1.057 | |  | |
| *se* | |  | | (0.0758) | |  | |  | | (0.0758) | |  | |
| *p-value* | |  | | 0.5023 | |  | |  | | 0.4418 | |  | |
| Income *(irr)*I | | 0.983 | |  | | 0.958 | | 0.999 | |  | | 0.976 | |
| *se* | | (0.0589) | |  | | (0.0589) | | (0.0611) | |  | | (0.0622) | |
| *p-value* | | 0.7686 | |  | | 0.4863 | | 0.9896 | |  | | 0.7053 | |
| Income² *(irr)* | |  | |  | | 1.039 | |  | |  | | 1.034 | |
| *se* | |  | |  | | (0.0271) | |  | |  | | (0.0272) | |
| *p-value* | |  | |  | | 0.1404 | |  | |  | | 0.2023 | |
|  | |  | |  | |  | |  | |  | |  | |
| N | | 828 | | 828 | | 828 | | 828 | | 828 | | 828 | |

*Notes:* All regressions are negative binomial models; the exponential of the estimated coefficient is reported, incident rate ratio, IRR. (a) Regressions include family income and a year dummy for 2008. (b) Regressions include family income, a year dummy for 2008, individual and family characteristics. Robust standard errors (s.e.) are in parentheses. Statistical significance levels: * p<=10%; ** p<=5%; *** p<=1%.

Table A10. Impact of CMUC eligibility on total conditional number of visits (Under 30)

|  | Without controls (a) | | | With controls (b) | | |
| --- | --- | --- | --- | --- | --- | --- |
|  | (1) | (2) | (3) | (1) | (2) | (3) |
|  |  |  |  |  |  |  |
| eliCMUCI *(irr)* | 1.514** | 1.455** | 1.450** | 1.503** | 1.443** | 1.438** |
| *se* | (0.2834) | (0.2758) | (0.2751) | (0.2761) | (0.2663) | (0.2656) |
| *p-value* | 0.0265 | 0.0479 | 0.0499 | 0.0267 | 0.0470 | 0.0491 |
| Eli*Income *(irr)* |  | 1.058 |  |  | 1.071 |  |
| *se* |  | (0.1406) |  |  | (0.1334) |  |
| *p-value* |  | 0.6699 |  |  | 0.5806 |  |
| Noeli*Income *(irr)* |  | 1.279** |  |  | 1.301** |  |
| *se* |  | (0.1337) |  |  | (0.1351) |  |
| *p-value* |  | 0.0184 |  |  | 0.0112 |  |
| Income *(irr)*I | 1.191** |  | 1.163* | 1.209** |  | 1.180** |
| *se* | (0.0983) |  | (0.0983) | (0.0972) |  | (0.0961) |
| *p-value* | 0.0340 |  | 0.0734 | 0.0183 |  | 0.0418 |
| Income² *(irr)* |  |  | 1.039 |  |  | 1.040 |
| *se* |  |  | (0.0372) |  |  | (0.0355) |
| *p-value* |  |  | 0.2802 |  |  | 0.2466 |
|  |  |  |  |  |  |  |
| N | 429 | 429 | 429 | 429 | 429 | 429 |

*Notes:* All regressions are negative binomial models; the exponential of the estimated coefficient is reported, incident rate ratio, IRR. (a) Regressions include family income and a year dummy for 2008. (b) Regressions include family income, a year dummy for 2008, individual and family characteristics. Robust standard errors (s.e.) are in parentheses. Statistical significance levels: * p<=10%; ** p<=5%; *** p<=1%.

*1.3.9 Probability of seeing at least once a specialist*

Table A11. Impact of CMUC eligibility on the probability of seeing a specialist (Full sample)

|  | Without controls (a) | | | | | | With controls (b) | | | | | |  |
| --- | --- | --- | --- | --- | --- | --- | --- | --- | --- | --- | --- | --- | --- |
|  | (1) | | (2) | | (3) | | (1) | | (2) | | (3) | |  |
|  |  | |  | |  | |  | |  | |  | |  |
| eliCMUCI *(od)* | | 1.168 | | 1.092 | | 1.118 | | 1.141 | | 1.055 | | 1.080 | |
| *se* | | (0.2772) | | (0.2727) | | (0.2790) | | (0.2762) | | (0.2683) | | (0.2748) | |
| *p-value* | | 0.5116 | | 0.7237 | | 0.6563 | | 0.5871 | | 0.8336 | | 0.7631 | |
| Eli*Income *(od)* | |  | | 0.880 | |  | |  | | 0.868 | |  | |
| *se* | |  | | (0.1434) | |  | |  | | (0.1443) | |  | |
| *p-value* | |  | | 0.4324 | |  | |  | | 0.3939 | |  | |
| Noeli*Income *(od)* | |  | | 1.056 | |  | |  | | 1.070 | |  | |
| *se* | |  | | (0.1242) | |  | |  | | (0.1289) | |  | |
| *p-value* | |  | | 0.6459 | |  | |  | | 0.5742 | |  | |
| Income *(od)* | | 0.992 | |  | | 0.974 | | 0.997 | |  | | 0.974 | |
| *se* | | (0.0948) | |  | | (0.0977) | | (0.0977) | |  | | (0.1003) | |
| *p-value* | | 0.9326 | |  | | 0.7901 | | 0.9749 | |  | | 0.7977 | |
| Income² *(od)* | |  | |  | | 1.025 | |  | |  | | 1.031 | |
| *se* | |  | |  | | (0.0432) | |  | |  | | (0.0442) | |
| *p-value* | |  | |  | | 0.5512 | |  | |  | | 0.4736 | |
|  | |  | |  | |  | |  | |  | |  | |
| N | | 2,232 | | 2,232 | | 2,232 | | 2,232 | | 2,232 | | 2,232 | |

*Notes:* All regressions are logit models; Odds ratio are reported (OR). (a) Regressions include family income and a year dummy for 2008. (b) Regressions include family income, a year dummy for 2008, individual and family characteristics. Robust standard errors (s.e.) are in parentheses. Statistical significance levels: * p<=10%; ** p<=5%; *** p<=1%.

Table A12. Impact of CMUC eligibility on the probability of seeing a specialist (Under 30)

|  | Without controls (a) | | | With controls (b) | | |
| --- | --- | --- | --- | --- | --- | --- |
|  | (1) | (2) | (3) | (1) | (2) | (3) |
|  |  |  |  |  |  |  |
| eliCMUCI *(od)* | 2.996** | 2.946** | 2.976** | 3.212** | 3.138** | 3.174** |
| *se* | (1.4074) | (1.4208) | (1.4402) | (1.5531) | (1.5534) | (1.5748) |
| *p-value* | 0.0195 | 0.0251 | 0.0242 | 0.0158 | 0.0209 | 0.0199 |
| Eli*Income *(od)* |  | 1.401 |  |  | 1.458 |  |
| *se* |  | (0.4708) |  |  | (0.4950) |  |
| *p-value* |  | 0.3161 |  |  | 0.2668 |  |
| Noeli*Income *(od)* |  | 1.496* |  |  | 1.598* |  |
| *se* |  | (0.3656) |  |  | (0.4026) |  |
| *p-value* |  | 0.0995 |  |  | 0.0628 |  |
| Income *(od)* | 1.461* |  | 1.456* | 1.547** |  | 1.537** |
| *se* | (0.2864) |  | (0.3006) | (0.3094) |  | (0.3216) |
| *p-value* | 0.0529 |  | 0.0687 | 0.0293 |  | 0.0400 |
| Income² *(od)* |  |  | 1.005 |  |  | 1.009 |
| *se* |  |  | (0.0887) |  |  | (0.0905) |
| *p-value* |  |  | 0.9535 |  |  | 0.9196 |
|  |  |  |  |  |  |  |
| N | 532 | 532 | 532 | 532 | 532 | 532 |

*Notes:* All regressions are logit models; Odds ratio are reported (OR). (a) Regressions include family income and a year dummy for 2008. (b) Regressions include family income, a year dummy for 2008, individual and family characteristics. Robust standard errors (s.e.) are in parentheses. Statistical significance levels: * p<=10%; ** p<=5%; *** p<=1%.

- 1. Absence of other discontinuity points

We checked the absence of other discontinuity points over the income distribution below and above the real eligibility cut-off point (${Cplacebo}_{t-1}$=6,500€ and 8,500€). Tables A13-14 report the linear probability model estimates of;

${CMUC}_{it}=\tilde{\alpha}+\tilde{\beta}*X_{it}+\tilde{\delta}*{eliCMUCplacebo}_{it}+{{eliCMUCplacebo}_{it}*\tilde{k}}_{g1}\left( Z_{it-1} \right)+{(1-{eliCMUCplacebo}_{it})*\tilde{k}}_{g2}\left( Z_{it-1} \right)+\tilde{u}_{it}$

where ${EliCMUCplacebo}_{it}=I\left( Z_{it-1}<{Cplacebo}_{t-1} \right)$, $X_{it}$ is a matrix of control variables, $\tilde{k}_{g1}$($Z_{it-1}$) and $\tilde{k}_{g1}$($Z_{it-1}$) are polynomial functions of income and, $\tilde{u}_{it}$ is the unobserved error component.

Table A13. Absence of other discontinuity points

in CMUC participation

| ${Cplacebo}_{t-1}$=6,500 Euros per CU *(on the left side of the eligibility threshold)* | | | |
| --- | --- | --- | --- |
| CMUC | Full | Under 30 | 30 & older |
| EliCMUCplacebo (1) | 0.066 | -0.052 | 0.107 |
|  | 0.0598 | 0.1294 | 0.0671 |
| EliCMUCplacebo (2) | 0.065 | -0.048 | 0.106 |
|  | 0.0606 | 0.1339 | 0.0683 |
| N | 639 | 167 | 472 |
| ${Cplacebo}_{t-1}$=8,500 Euros per CU *(on the right side of the eligibility threshold)* | | | |
|  | Full | Under 30 | 30 & older |
| EliCMUCplacebo (1) | 0.001 | 0.035 | -0.008 |
|  | 0.0147 | 0.0316 | 0.0168 |
| EliCMUCplacebo (2) | 0.001 | 0.034 | -0.007 |
|  | 0.0146 | 0.0316 | 0.0165 |
| N | 1,593 | 365 | 1,228 |

*Notes:* (1) All regressions are linear probability models including family income and a year dummy for 2008. (2) All regressions are linear probability models including family income, a year dummy for 2008, individual and family characteristics. Robust standard errors are in parentheses. Statistical significance levels: * p<=10%; ** p<=5%; *** p<=1%.

Table A14. Absence of other discontinuity points

in the proportion of no-coverage

| ${Cplacebo}_{t-1}$=6,500 Euros per CU *(on the left side of the eligibility threshold)* | | | |
| --- | --- | --- | --- |
| CMUC | Full | Under 30 | 30 & older |
| EliCMUCplacebo (1) | 0.113 | 0.184 | 0.081 |
|  | 0.0711 | 0.1324 | 0.0839 |
| EliCMUCplacebo (2) | 0.096 | 0.168 | 0.064 |
|  | 0.0714 | 0.1358 | 0.0836 |
| N | 639 | 167 | 472 |
| ${Cplacebo}_{t-1}$=8,500 Euros per CU *(on the right side of the eligibility threshold)* | | | |
|  | Full | Under 30 | 30 & older |
| EliCMUCplacebo (1) | 0.009 | 0.019 | 0.013 |
|  | 0.0404 | 0.0889 | 0.0453 |
| EliCMUCplacebo (2) | 0.014 | 0.016 | 0.015 |
|  | 0.0402 | 0.0886 | 0.0452 |
| N | 1,593 | 365 | 1,228 |

*Notes:* (1) All regressions are linear probability models including family income and a year dummy for 2008. (2) All regressions are linear probability models including family income, a year dummy for 2008, individual and family characteristics. Robust standard errors are in parentheses. Statistical significance levels: * p<=10%; ** p<=5%; *** p<=1%.
